# Supplementary material for: Interventions to improve the mental health of women experiencing homelessness: A systematic review of the literature
Source: PLoS One. 2024 Apr 3;19(4):e0297865. doi: 10.1371/journal.pone.0297865 (PMC10990227; doi:10.1371/journal.pone.0297865)
Supplement: S1 File — (PDF) [file pone.0297865.s002.pdf]

Appendix: Search strategies for different databases

Embase

1. exp homeless person/ or exp homelessness/
2. *"displacement (people)"/
3. (homeless* or hostel* or shelter*).ab. or (homeless* or hostel* or shelter*).ti.
4. ((temporar* or emergency or vulnerabl*) adj2 (accommodat* or hous*)).ab. or ((temporar* or emergency or vulnerabl*) adj2 (accommodat* or hous*)).ti.
5. ("rough sleep*" or runaway).ab. or ("rough sleep*" or runaway).ti.
6. 1 or 2 or 3 or 4 or 5
7. *female/ or mother/ or *adolescent mother/ or *expectant mother/
8. (wom#n or girl* or female* or mother*).ab. or (wom#n or girl* or female* or mother*).ti.
9. 7 or 8
10. mental disease/ or addiction/ or adjustment disorder/ or anxiety disorder/ or autism/ or behavior disorder/ or complicated grief/ or delirium/ or dissociative disorder/ or emotional disorder/ or mental instability/ or mood disorder/ or neurosis/ or personality disorder/ or psychosexual disorder/ or psychosis/ or psychosomatic disorder/ or psychotrauma/ or schizophrenia spectrum disorder/
11. exp automutilation/di, dm, ep, pc, rh, th [Diagnosis, Disease Management, Epidemiology, Prevention, Rehabilitation, Therapy]
12. exp suicide/di, dm, ep, pc, rh, th [Diagnosis, Disease Management, Epidemiology, Prevention, Rehabilitation, Therapy]
13. "mental health".ab. or "mental health”.ti. or ((psych* or mental*) adj2 (ill* or disorder* or disease* or problem*)).ab. or ((psych* or mental*) adj2 (ill* or disorder* or disease* or problem*)).ti.
14. ("mood disorder*" or addict* or depress* or bipolar or schizo* or "post-traumatic stress" or "PTSD" or anxiety).ab. or ("mood disorder*" or addict* or depress* or bipolar or schizo* or "post-traumatic stress" or "PTSD" or anxiety).ti.
15. ("self-harm*" or "self-injur*" or suicid*).ab. or ("self-harm*" or "self-injur*" or suicid*).ti. or ((drug* or alcohol or substance*) and (abus* or misus* or us* or dependen*)).ab. or ((drug* or alcohol or substance*) and (abus* or misus* or us* or dependen*)).ti.
16. 10 or 11 or 12 or 13 or 14 or 15
17. *therapy/ or *health program/ or exp psychosocial intervention/ or *mental health service/ or exp community mental health service/ or *primary prevention/ or *secondary prevention/ or *integrated health care system/

18. (intervention* or program* or support* or treatment* or strateg* or education* or therap*).ab. or (intervention* or program* or support* or treatment* or strateg* or education* or therap*).ti.
19. 17 or 18
20. 6 and 9 and 16 and 19

Medline via Ovid

1. exp Homeless Youth/ or exp Homeless Persons/
2. (homeless* or hostel* or shelter*).ab. or (homeless* or hostel* or shelter*).ti.
3. ((temporar* or emergency or vulnerabl*) adj2 (accommodat* or hous*)).ab. or ((temporar* or emergency or vulnerabl*) adj2 (accommodat* or hous*)).ti.
4. ("rough sleep*" or runaway).ab. or ("rough sleep*" or runaway).ti.
5. 1 or 2 or 3 or 4
6. women/ or battered women/ or pregnant women/ or mothers/
7. (wom#n or girl* or female* or mother*).ab. or (wom#n or girl* or female* or mother*).ti.
8. 6 or 7
9. mental disorders/ or anxiety disorders/ or "bipolar and related disorders"/ or "disruptive, impulse control, and conduct disorders"/ or dissociative disorders/ or "feeding and eating disorders"/ or mood disorders/ or exp depressive disorder/ or cyclothymic disorder/ or neurodevelopmental disorders/ or neurotic disorders/ or personality disorders/ or "schizophrenia spectrum and other psychotic disorders"/ or sexual dysfunctions, psychological/ or sleep wake disorders/ or somatoform disorders/ or substance-related disorders/ or "trauma and stressor related disorders"/ or self-injurious behavior/ or self mutilation/ or suicide/
10. "mental health".ab. or "mental health".ti. or ((psych* or mental*) adj2 (ill* or disorder* or disease* or problem*)).ab. or ((psych* or mental*) adj2 (ill* or disorder* or disease* or problem*)).ti.
11. ("mood disorder*" or addict* or depress* or bipolar or schizo* or "post-traumatic stress" or "PTSD" or anxiety).ab. or ("mood disorder*" or addict* or depress* or bipolar or schizo* or "post-traumatic stress" or "PTSD" or anxiety).ti.
12. ("self-harm*" or "self-injur*" or suicid*).ab. or ("self-harm*" or "self-injur*" or suicid*).ti. or ((drug* or alcohol or substance*) and (abus* or misus* or us* or dependen*)).ab. or ((drug* or alcohol or substance*) and (abus* or misus* or us* or dependen*)).ti.
13. 9 or 10 or 11 or 12
14. Crisis Intervention/ or exp Psychotherapy/mt, tu [Methods, Therapeutic Use] or exp Mental Health Services/ or "Delivery of Health Care, Integrated"/

15. (intervention* or program* or support* or treatment* or strateg* or education* or therap*).ab. or (intervention* or program* or support* or treatment* or strateg* or education* or therap*).ti.
16. 14 or 15
17. 5 or 8 or 13 or 16

PsycINFO via EBSCO

1. DE "Homeless" OR DE "Social Issues" OR DE "Homeless Mentally Ill" OR DE "Shelters"
2. TI (homeless* or hostel* or shelter*) OR AB (homeless* or hostel* or shelter*)
3. TI ((temporar* or emergency or vulnerabl*) N2 (accommodat* or hous*)) OR AB ((temporar* or emergency or vulnerabl*) N2 (accommodat* or hous*))
4. TI ("rough sleep*" or runaway) OR AB ("rough sleep*" or runaway)
5. S1 OR S2 OR S3 OR S4
6. DE "Human Females" OR DE "Battered Females" OR DE "Mothers" OR DE "Widows" OR DE "Wives" OR DE "Female Delinquency"
7. TI (wom#n or girl* or female* or mother*) OR AB (wom#n or girl* or female* or mother*)
8. S6 OR S7
9. DE "Mental Disorders" OR DE "Affective Disorders" OR DE "Anxiety Disorders" OR DE "Autism Spectrum Disorders" OR DE "Bipolar Disorder" OR DE "Borderline States" OR DE "Chronic Mental Illness" OR DE "Dissociative Disorders" OR DE "Eating Disorders" OR DE "Gender Dysphoria" OR DE "Neurocognitive Disorders" OR DE "Neurodevelopmental Disorders" OR DE "Neurosis" OR DE "Personality Disorders" OR DE "Psychosis" OR DE "Serious Mental Illness" OR DE "Somatoform Disorders" OR DE "Stress and Trauma Related Disorders" OR DE "Substance Related and Addictive Disorders"
10. DE "Self-Injurious Behavior" OR DE "Self-Destructive Behavior" OR DE "Head Banging" OR DE "Self-Inflicted Wounds" OR DE "Self-Mutilation" OR DE "Self-Poisoning" OR DE "Suicide"
11. TI ("mental health" or ((psych* or mental*) N2 (ill* or disorder* or disease* or problem*))) OR AB ("mental health" or ((psych* or mental*) N2 (ill* or disorder* or disease* or problem*)))
12. TI ("mood disorder*" or addict* or depress* or bipolar or schizo* or "post-traumatic stress" or PTSD or anxiety) OR AB ("mood disorder*" or addict* or depress* or bipolar or schizo* or "post-traumatic stress" or PTSD or anxiety)

13. TI (((drug* or alcohol or substance*) and (abus* or misus* or us* or dependen*))) OR AB (((drug* or alcohol or substance) and (abus* or misus* or us* or dependen*)))
14. TI ("self-harm*" or "self-injur*" or suicid*) OR AB ("self-harm*" or "self-injur*" or suicid*)
15. S9 OR S10 OR S11 OR S12 OR S13 OR S14
16. DE "Intervention" OR DE "Treatment" OR DE "Crisis Intervention" OR DE "Early Intervention" OR DE "Family Intervention" OR DE "Group Intervention" OR DE "At Risk Populations" OR DE "Crisis Intervention Services" OR DE "Prevention" OR DE "Rehabilitation" OR DE "Crisis Intervention Services" OR DE "Suicide Prevention Centers" OR DE "Community Mental Health" OR DE "Community Mental Health Centers" OR DE "Community Mental Health Services" OR DE "Community Psychiatry" OR DE "Community Psychology" OR DE "Integrated Services" OR DE "Outreach Programs" OR DE "Preventive Mental Health Services" OR DE "Public Health Services"
17. TI (intervention* or program* or support* or treatment* or strateg* or education* or therap*) OR AB (intervention* or program* or support* or tool* or training* or treatment* or strateg* or education* or therapy)

ASSIA via Proquest

1. MAINSUBJECT.EXACT("Homeless mentally ill young people") OR MAINSUBJECT.EXACT("Homeless people") OR MAINSUBJECT.EXACT("Homeless young people") OR MAINSUBJECT.EXACT("Homeless mentally ill people") OR MAINSUBJECT.EXACT("Homeless adolescent girls") OR MAINSUBJECT.EXACT("Homelessness") OR MAINSUBJECT.EXACT("Homeless mentally ill women") OR MAINSUBJECT.EXACT("Homeless mothers") OR MAINSUBJECT.EXACT("Homeless families") OR MAINSUBJECT.EXACT("Homeless women") OR MAINSUBJECT.EXACT("Homeless young women") OR MAINSUBJECT.EXACT("Homeless pregnant women")
2. ab(homeless* or hostel* or shelter*) OR ti(homeless* or hostel* or shelter*)
3. ab((temporar* or emergency or vulnerabl*) near/2 (accommodat* or hous*)) OR ti((temporar* or emergency or vulnerabl*) near/2 (accommodat* or hous*))
4. ab((“rough sleep*” or runaway)) OR ti((“rough sleep*” or runaway))
5. 1 AND 2 AND 3 AND 4
6. MAINSUBJECT.EXACT("Emotionally disturbed women") OR MAINSUBJECT.EXACT("Poor women") OR MAINSUBJECT.EXACT("Disadvantaged women") OR MAINSUBJECT.EXACT("Battered women") OR

MAINSUBJECT.EXACT("Elderly women") OR MAINSUBJECT.EXACT("Disabled women") OR MAINSUBJECT.EXACT("Low income women") OR MAINSUBJECT.EXACT("Poor pregnant women") OR MAINSUBJECT.EXACT("Women") OR MAINSUBJECT.EXACT("Mentally ill women") OR MAINSUBJECT.EXACT("Pregnant women")

7. ab((wom#n or girl* or female* or mother*)) OR ti((wom#n or girl* or female* or mother*))
8. 6 OR 7
9. MAINSUBJECT.EXACT("Delusional disorders") OR MAINSUBJECT.EXACT("Personality disorders") OR MAINSUBJECT.EXACT("Chronic psychiatric disorders") OR MAINSUBJECT.EXACT("Behaviour disorders") OR MAINSUBJECT.EXACT("Emotional disorders") OR MAINSUBJECT.EXACT("Attachment disorders") OR MAINSUBJECT.EXACT("Mania") OR MAINSUBJECT.EXACT("Affective disorders") OR MAINSUBJECT.EXACT("Somatoform disorders") OR MAINSUBJECT.EXACT("Psychiatric disorders") OR MAINSUBJECT.EXACT("Psychoses") OR MAINSUBJECT.EXACT("Anxiety disorders") OR MAINSUBJECT.EXACT("Schizoaffective disorder") OR MAINSUBJECT.EXACT("Psychotic mood disorders") OR MAINSUBJECT.EXACT("Adjustment disorder") OR MAINSUBJECT.EXACT("Depression") OR MAINSUBJECT.EXACT("Schizophrenia") OR MAINSUBJECT.EXACT("Mental illness")
10. MAINSUBJECT.EXACT("Alcohol misuse") OR MAINSUBJECT.EXACT("Drug addiction") OR MAINSUBJECT.EXACT("Addiction") OR MAINSUBJECT.EXACT("Intravenous drug addiction") OR MAINSUBJECT.EXACT("Alcohol dependence")
11. MAINSUBJECT.EXACT("Suicide") OR MAINSUBJECT.EXACT("Selfpoisoning") OR MAINSUBJECT.EXACT("Selfinjury")
12. ab(("mental health" or ((psych* or mental*) near/2 (ill* or disorder* or disease* or problem*)))) OR ti(("mental health" or ((psych* or mental*) near/2 (ill* or disorder* or disease* or problem*))))
13. ab("mood disorder*" or addict* or depress* or bipolar or schizo* or "post-traumatic stress" or PTSD or anxiety) OR ti("mood disorder*" or addict* or depress* or bipolar or schizo* or "post-traumatic stress" or PTSD or anxiety)
14. 9 OR 10 OR 11 OR 12 OR 13

15. MAINSUBJECT.EXACT("Early intervention programmes") OR MAINSUBJECT.EXACT("Brief interventions") OR MAINSUBJECT.EXACT("State intervention") OR MAINSUBJECT.EXACT("Psychosocial intervention") OR MAINSUBJECT.EXACT("Social interventions") OR MAINSUBJECT.EXACT("Psychological intervention") OR MAINSUBJECT.EXACT("Intervention") OR MAINSUBJECT.EXACT("Interventions") OR MAINSUBJECT.EXACT("Crisis intervention")
16. MAINSUBJECT.EXACT("Community psychology") OR MAINSUBJECT.EXACT("Preventive programmes") OR MAINSUBJECT.EXACT("Community mental health services") OR MAINSUBJECT.EXACT("Community support programmes") OR MAINSUBJECT.EXACT("Housing assistance programmes") OR MAINSUBJECT.EXACT("Outreach programmes") OR MAINSUBJECT.EXACT("Community psychiatric nursing") OR MAINSUBJECT.EXACT("Integrated services")
17. ab(intervention* or program* or support* or treatment* or strateg* or education* or therap*) OR ti(intervention* or program* or support* or treatment* or strateg* or education* or therap*)
18. 15 OR 16 OR 17
19. 5 AND 8 AND 14 AND 18

CINAHL via OpenAthens

1. (MH "Homeless Persons") OR (MH "Homelessness")
2. AB (homeless* or hostel* or shelter*) OR TI (homeless* or hostel* or shelter*)
3. AB ((temporar* or emergency or vulnerabl*) N2 (accommodat* or hous*)) OR TI ((temporar* or emergency or vulnerabl*) N2 (accommodat* or hous*))
4. AB (“rough sleep*” or runaway) OR TI (“rough sleep*” or runaway)
5. S1 OR S2 OR S3 OR S4
6. (MH "Women") OR (MH "Battered Women") OR (MH "Expectant Mothers")
7. TI (wom#n or girl* or female* or mother*) OR AB (wom#n or girl* or female* or mother*)
8. S6 or S7
9. (MH "Mental Disorders") OR (MH "Depression+") OR (MH "Anxiety Disorders") OR (MH "Personality Disorders") OR (MH "Psychotic Disorders") OR (MH "Substance Use Disorders") OR (MH "Alcohol-Related Disorders+") OR (MH "Substance misuse+") OR (MH "Substance Dependence+") OR (MH "Mental Disorders, Chronic") OR (MH "Organic Mental Disorders, Substance-Induced") OR (MH "Organic Mental Disorders, Psychotic") OR (MH "Substance Dependence") OR (MH "Alcoholism")

10. (MH "Injuries, Self-Inflicted") OR (MH "Self-Injurious Behavior")
11. (MH "Suicide") OR (MH "Suicide, Attempted") OR (MH "Suicidal Ideation")
12. TI ("mental health" or ((psych* or mental*) N2 (ill* or disorder* or disease* or problem*))) OR AB ("mental health" or ((psych* or mental*) N2 (ill* or disorder* or disease* or problem*)))
13. TI ("mood disorder*" or addict* or depress* or bipolar or schizo* or "post-traumatic stress" or PTSD or anxiety) OR AB ("mood disorder*" or addict* or depress* or bipolar or schizo* or "post-traumatic stress" or PTSD or anxiety)
14. TI (((drug* or alcohol or substance*) and (abus* or misus* or us* or dependen*))) OR AB (((drug* or alcohol or substance*) and (abus* or misus* or us* or dependen*)))
15. TI ("self-harm*" or "self-injur*" or suicid*) OR AB ("self-harm*" or "self-injur*" or suicid*)
16. S9 OR S10 OR S11 OR S12 OR S13 OR S14 OR S15 OR S16
17. (MH "Psychosocial Intervention") OR (MH "Early Intervention") OR (MH "Intervention Trials") OR (MH "Nursing Interventions") OR (MH "Crisis Intervention") OR (MH "Crisis Intervention (Iowa NIC)")
18. (MH "Suicide Prevention (Iowa NIC)") OR (MH "Substance Use Prevention (Iowa NIC)")
19. (MH "Community Mental Health Services") OR (MH "Community Mental Health Nursing") OR (MH "Community Health Centers")
20. TI (intervention* or program* or support* or treatment* or strateg* or education* or therap*) OR AB (intervention* or program* or support* or treatment* or strateg* or education* or therap*)
21. S17 OR S18 OR S19 OR S20
22. S5 AND S8 AND S16 AND S2
