# Supplementary material for: Interventions to improve the mental health of women experiencing homelessness: A systematic review of the literature
Source: PLoS One. 2024 Apr 3;19(4):e0297865. doi: 10.1371/journal.pone.0297865 (PMC10990227; doi:10.1371/journal.pone.0297865)
Supplement: S2 File — (PDF) [file pone.0297865.s003.pdf]

Author Year Country<br><br>Study Design	Study aims	Control condition	Setting	Sample size and characteristics	Outcome measures	Findings Q1	Findings Q2
Bain 2014 South Africa<br><br>RCT	This study aimed to explore the use of the manualized New-Beginnings parent–infant psychotherapy group model in an African setting with high-risk mother–infant dyads in homeless shelters. The outcomes were maternal reflexive function, maternal depression and anxiety, mother's sensitivity and infant's level of responsiveness, infant development	Women in the control group received shelter services as normal and then received the intervention at a later date	2 homeless shelters	22 homeless mothers<br><br>The mother–infant dyads who participated in this study were all homeless and had been living at one of the two shelters for 2 months to 2 years. At pretesting, the mothers ranged in age from 18 to 43, and the babies ranged in age from 9 days to 2½ years. Only 7 of the 22 mothers had completed their schooling; of those who had completed, only 4 women had any tertiary qualifications. All mothers were Black African ethnicity.	Q1: Depression and anxiety. Mothers' levels of depression and anxiety were measured using the Kessler-10 scale. The Kessler-10 is a 10-item self-report questionnaire intended to yield a global measure of "psychological distress" based on questions about the level of anxiety and depressive symptoms in the most recent 4-week period. According to Hides et al. (2007), individuals with a positive score on the Kessler-10 were 10 times more likely to have a current affective disorder. A recent South African study also has found the Kessler-10 to display good sensitivity and specificity in detecting depression (0.66), posttraumatic stress disorder (0.69), panic disorder (0.71), and social phobia (0.76), and has suggested that it may be a useful screening measure for mood and anxiety disorders in South African pregnant	Maternal Depression and Anxiety Levels, Mothers' results on pretesting reflected their traumatic early histories and current situations of deprivation and helplessness. On the Kessler-10, all mothers reported moderate to severe clinical symptoms of anxiety and depression and should have been accessing treatment (All scores were >16, with 36% of the mother falling into the high-risk range for anxiety and depressive disorders.)While the program was not aimed at alleviating maternal depression, note that levels of depression and anxiety appeared to increase slightly across all groups, with 72% of mothers reporting slightly higher symptom levels at post testing.	NA

					women (Spies et al., 2009).<br><br>Q2: N/A		

Bani-Fatemi et al. 2020 Canada<br><br>Cohort (one group pre and post)	Quantitatively evaluate changes in mental health and well-being outcomes in female identified youth experiencing Gender based violence and homelessness, 12 months after enrolment in a community-based, trauma-informed, brief group psychoeducation intervention.	No control group: outcome measures after intervention were compared to outcome measures before intervention in same women	Community resource centre for homeless youth with on-site crisis shelter	70 women<br><br>The mean age of participants at enrolment was 21.47 years (SD: 3.79). Nearly half of the participants (48.6%) were born in Canada. Over a third of participants were black (37.2%), while 22.8% were white and 40% reported other ethnicities; Two individuals (2.8%) reported being married, while 11 participants (16%) reported having children. Two of the participants (2.8%) identified as transgender, one participant disclosed her gender as both female and male, and one participant did not disclose their gender. Nearly half (45.7%) of the participants did not complete high school, 24.3% completed high school, and 30% had some postsecondary education. The average total Adverse Childhood Events (ACE) score among participants was 6.14 (SD: 2.90), with most participants (82.9%) having a total ACE score of 4 or more,	Q1: Relevant outcome measures to the study question: 1. 1. WHO Quality of Life, Shortened Version (WHOQOL-BREF) - Psychological health domain.<br>2. Hospital Anxiety and Depression Scale (HADS). Scores range from0 to 21 for each subscale with higher scores suggesting higher severity of anxiety or depression.<br>3. Global Assessment of Individual Need—Substance Problem Scale (GAIN-SPS)<br>4. UCLA-PTSD Reaction Index<br><br>Q2:N/A	WHO QOL Standardised survey (Psychological health domain) Mean score at baseline (SD): 12.36 (3.47), Mean score at 6 months (SD): 13.39 (3.43), Absolute Difference from baseline (95% CI): −1.032 (-2.512 to 0.447), p value: 0.28, Mean score at 12 months (SD): 13.37 (3.19), Difference from baseline (95% CI): −1.010 (-2.505 to 0.486), p-value: 0.31, overall p-value: 0.15 - not significant<br>HADS – A Scale: Mean score at baseline (SD): 10.86 (4.00), Mean score at 6 months (SD): 9.65 (4.63), Absolute difference at 6 months (95% CI): 1.209 (-0.635 to 3.053), p-value: 0.34, Mean score at 12 months (SD): 10.06 (4.02), Absolute difference at 12 months (95% CI): 0.799 (-1.064 to 2.663), p-value: 0.90, overall p-value: 0.27.<br>HADS – D Scale: Mean at baseline (SD): 7.04 (3.95), Mean at 6 months (SD): 6.24 (3.10), Absolute difference at 6 months (95% CI): 0.802 (-0.778 to 2.383), p-value: 0.66 , Mean at 12 months (SD): 5.96 (3.62), Absolute difference at 12 months (95% CI): 1.158 (-0.439 to 2.756), p-value: 0.24,	NA

				and only 4.3% reporting no adverse childhood experiences. The most common ACE reported was emotional abuse (75.7%), while physical neglect was the least commonly reported ACE (34.3%). The most common type of GBV described was family violence, reported by 64% of the participants. Many of our participants (n = 45 or 64.3%) experienced more than one type of GBV, while three of the participants (4.3%) did not disclose the type of violence experienced.		overall p-value: 0.19. UCLA-PTSD Scale: Mean at baseline (SD): 58.84 (25.99), Mean at 6-months: 49.61 (26.38), Absolute difference at 6 months (95% CI): 9.232 (-2.455 to 20.919), p-value: 0.17, Mean at 12 months (SD): 51.63 (27.91), Absolute difference at 12 months (95% CI): 7.208 (-4.605 to 19.022), p-value: 0.43, overall p-value: 0.13.	
Castanos-Cervantes et al 2019 Mexico<br><br>Matched pairs design (unclear if pairs were randomised)	The aims of this study were to (a) assess the preliminary effect of a brief cognitive behavioural group intervention in the well-being of a group of Mexican homeless girls, (b) examine the preliminary efficacy of the intervention for homeless girls with symptoms of anxiety and depression, and (c) determine the clinical significance of the treatment effect.	Girls receiving services from homeless youth and children NGOs who agreed to reside at selected shelters for the study period but did not receive the intervention. Some of these girls who were identified during their shelter stay as having severe mental health problems received individual therapy, if that shelter had a therapist available. Some of these girls would have attended	Homeless shelters across Mexico City	84 homeless girls<br><br>Girls ranged in age from 9-17. No further demographic information.	Q1: Symptoms of anxiety scale, symptoms of depression scale, assertive skills scale, emotion regulation scale, subjective wellbeing scale. Scales were developed by the authors based on items from various Mexican scales for children and youth that assess anxiety, depression, assertiveness, emotion regulation, and subjective well-being. The scales were	After the CBT intervention (post-test), Student's t-test showed significant differences in symptoms of anxiety and depression, assertiveness, subjective well-being, and emotion regulation strategies between the CBT group and TAU group. CBT treatment effects ranged from moderate to high according to Cohen's d for independent samples. The difference between pre-test and post-test scores for each treatment group was analysed separately with a	NA

		shelter workshops, but not CBT.			validated in a series of 3 pilot studies.<br><br>Q2: N/A	Student's t-test for paired samples. In the case of the CBT group, Student's t-test for paired samples revealed significant differences in symptoms of anxiety and depression, assertiveness, emotion regulation strategies and subjective well-being. Treatment effects ranged from moderate to high using Cohen's d. In the TAU group, Student's t-test for paired samples showed no significant differences in any variable. Treatment effects were low using Cohen's d.	
Constantino et al. 2005 USA<br><br>Pilot block RCT	The purpose of this pilot study was to test the feasibility of an intervention comparing post treatment improvement in health outcomes between subjects in the Social Support Intervention (SSI) group and No Treatment Control (NTC) group. The three research questions in this study are: (1) Does the SSI group show greater improvement in psychological distress symptoms as measured by the Brief Symptom Inventory (BSI) than the NTC group post treatment? (2) Does the SSI group show greater	Women shelter residents who attended a free-flowing chat session in place of the SS intervention	Domestic violence shelter	24 women<br><br>Most women were white (70.8%, n = 17) not Hispanic, and the rest were African Americans. The women were from 28 to 43 years of age. Most of the women completed high school, and three women had college degrees.	Q1: Psychological distress measured using the brief symptom inventory (BSI). Social support measured using the Interpersonal Self-evaluation List (ISEL). Health care utilisation and general health measured using the Health Screening Questionnaire (HSQ).<br><br>Q2: N/A	Group comparison results showed that the SSI group had greater improvement (p=.013) (Table 2) in psychological distress symptoms as measured by the BSI than the NTC group post treatment. Specifically, the SSI group showed pre to post intervention BSI means of 152.15 to 108.38, while the NTC group showed pre to post BSI means of 159.73 to 151.36	NA

	improvement in perceived availability of social support as measured by the Interpersonal Self Evaluation List (ISEL) than the NTC group post treatment? and (3) Does the SSI group show less health care utilization as measured by the Health Screening Questionnaire (HSQ) than the NTC group post treatment?						
Desai et al. 2008 USA<br><br>Cohort (2 groups pre and post; non-randomised trial)	Assess the effectiveness of the Seeking Safety programme in improving the mental and physical health of homeless female veterans	Conducted in advance of SS (Phase II), the control group, or Phase I involved no specific intervention other than case management, assessment of the specific needs of clients and referral for treatment, and time-limited transitional residential treatment available through the Homeless Women Veterans Programs.	Veteran's Affairs Medical Centres	450 women. 359 in control group, 91 in SS group<br><br>TAU clients were significantly younger than SS clients. SS clients were significantly less likely to have been employed full-time for most of the previous three years and more likely to have been unemployed. There were no baseline differences on other demographic characteristics.	Q1: Relevant outcome measures: Addiction Severity Index (ASI), 30-item Symptom Checklist Revised (SCL-30-R) for mental health screening, 12-Item Short Form Survey (12-SF) for physical and mental functioning, PTSD Checklist.<br><br>Q2: N/A	SS group had significantly better outcomes than those in TAU on SCL-30-R symptom scores (p=.03); PTSD Checklist scores (p=.03), particularly on the arousal (p=.04) and avoidant (p=.007) subscales and the ASI psychiatric composite (p=.03). In addition, there were significant interactions between group and time for social support (p=.04), SCL-30-R scores (p=.04), and PTSD Checklist scores (p=.04), particularly on the arousal (p=.02) and avoidant(p=.03) subscales. The interactions indicated greater rates of	NA

						improvement for SS clients. However, Seeking Safety clients were significantly more likely to have used drugs in the past 30 days (p=.04).	

Grabbe et al. 2013 USA<br><br>Qualitative descriptive design with a conventional content analysis of narrative data.	This pilot project aimed to explore gardening as a means of promoting positive mental health at a large daytime shelter for homeless women. Women's attitudes towards the intervention were determined in this qualitative study, which reflected the acceptability of the intervention.	No control group	Daytime homeless centre	Q1: The 8 women who were interviewed ranged in age from 20 to 59 years, with a median age of 50.<br>Four of the women had a 12th-grade education or less, and 4 had had some college or technical school.<br>Four of the 8 participants reported having one or more mental illnesses; these included bipolar disorder, depression, obsessive-compulsive disorder, and borderline personality disorder.<br><br>Q2: N/A	Q1: NA<br><br>Q2: Open-ended interviews were conducted with eight women who had attended at least eight gardening sessions. An interview guide with broad questions and follow-up probes was used to explore the women's perceptions of the gardening activity, including what the garden meant to them and how they felt while gardening.	NA	Attitudes to the gardening activity fell into 3 main themes: stress relief, social inclusion and personal change.<br>Stress relief: Feeling stress relief. The physical work of the garden was a distraction from the many stresses of homelessness, and the tangible results of gardening provided a psychological boost. The women watched the vegetables and herbs grow and regularly ate the produce. Most of the women spoke of the garden as relaxing, positive, and therapeutic: "It clears your mind . . .," "It's a way to escape. . ." and one woman explained to us: "It definitely changes the rest of my day. . . . the fact that I know that I'm going to be here doing this helps a lot."<br>Taking care of plants interrupted the cycle of depression and hopelessness for some. One woman reported, "I feel lonely sometimes and depressed a lot, but since I've been in the garden I don't feel as bad. I still have my depression but I don't get as bad."<br>Social inclusion: Feeling socially included. Gardening, exercising outdoors, and preparing and talking about food

							and recipes are unremarkable activities for housed individuals. For our participants, these simple and ordinary activities were a departure from shelter existence, allowing them to feel more themselves and therefore less marginalized<br>Personal change. The narratives of the women touched on multiple aspects of personal growth or change: taking risks, accepting failure, learning/ teaching, and tolerance of others. The garden effort, like the women's very existence, required tenacity to keep going even when things did not turn out as desired.
Graziano et al2023<br>USA<br><br>RCT	To assess the feasibility and acceptability of time-limited adaptations of parent–child interaction therapy (PCIT) and child–parent psychotherapy (CPP) for children experiencing homelessness	No control group, two experimental conditions	Homeless shelter	Sample included 144 children whose mothers consented to take part in the study and 144 mothers.<br><br>Children's mean age was 3.48 years, 43.1% were females, 78.5% of children were Black/African American. One child was on psychotropic medication. Maternal sample was also 144; 71,5% of mothers were unemployed, nearly 40% experienced at least one form of abuse in the past.	Q1: 1. Parenting stress measured using Dyadic Parent–Child Interaction Coding System–4th Edition<br>2. Child externalising behaviours measured with Eyberg Child Behavior Inventory (ECBI)<br>3. Posttraumatic stress symptoms measured with the Child and Adolescent Trauma Screen– Caregiver (CATS-C)<br><br>Q2: Acceptability and feasibility measured by intervention completion and	Mothers in both time-limited PCIT and time-limited CPP reported significant reductions in terms of their parenting stress and significant improvements in proportion of positive parenting verbalizations. Only mothers in time-limited PCIT reported significant reductions in negative parenting verbalizations. Mothers in both interventions reported significant reductions in their children's PTSS. Only mothers in time-limited PCIT noted significant reductions in their	14.9% of families in time-limited CPP and 8.6% of families in time-limited PCIT dropped out after randomization. Of families that initiated intervention, 48.4% of families in time-limited PCIT (n = 31) completed the intervention within 16 weeks compared to 39.7% of families in time-limited CPP (n = 25). The difference was not statistically significant. 28% of families completed at least 10 sessions within 12 weeks, 53% of completed at least 10 sessions within 14 weeks, 65% of families

					attendance, and consumer satisfaction	children's externalizing behavior problems.	completed at least 10 sessions within 16 weeks, 85% of families were able to complete at least 10 sessions within 20 weeks, and 93% of families were able to complete at least 10 sessions within 24 weeks. The average number of attended sessions did not differ significantly between groups. Mothers reported high levels of overall satisfaction across both interventions noting greatest improvements in their parent–child relationship, feeling like their child made progress in terms of their general behavior, progress related to their trauma symptoms or traumatic/stressful experiences, and generally positive feelings about the parenting programs. over 90% indicated that they would likely recommend both programs to others.

Guo et al. 2012 USA<br><br>RCT	Assess maternal and child mental health outcomes among homeless mothers receiving housing and supportive services (Ecologically-Based Treatment, EBT) versus mothers receiving community-based housing and support services (treatment as usual, TAU).	TAU included emergency shelter for women and their children up to 3 weeks at the shelter and linkage to housing and support services in the community. The shelter follows a rapid-rehousing approach, and is considered a national model for ending homelessness among families. Otherwise, the shelter provides 3 months of subsidized housing with the expectation that women will secure employment within that time frame and become responsible for the rent.	Community housing	60 women recruited from homeless shelters. 30 control group, 30 experimental group.<br><br>Homeless mothers in the current sample were 26.30 years old on average. Forty-five (75 %) of the mothers were African-American. The average number of children that each mother had was 2.82, ranging in age from 1 to 8 years (SD = 3.34). The average age of the target children was 3.68 years, and 29 children were female (48.3 %).	Q1: 1. The Computerized Diagnostic Interview Schedule for the DSM-IV (Robins et al. 2000) was used to screen mothers for substance abuse or dependence<br>2. The Short-Form-36 (SF-36) was utilized as a general assessment of health status of the homeless mothers (contained MH subscale)<br>3. The Beck Depression Inventory-II (BDI-II; Beck et al. 1996) was administered to assess mothers' depressive symptoms.<br><br>Q2: N/A	Mothers in both treatment conditions showed significant improvements in their depressive symptoms, mental health (all p's < 0.05, see Table 3) across time. None of the treatment condition differences were significant (all p's >0.05).	NA

Harpaz-Rotem 2011 USA<br><br>Cohort study	Assess the effectiveness of >30 days of Residential Treatment (RT) in Veteran's Affairs Community treatment units in improving mental, physical and social outcomes in homeless female veterans with psychiatric or substance use disorders	Homeless female veterans accessing the VA medical centres who agreed to participate in the study and did not make use of RT for >30 days	Community residential treatment centres run by Veteran's Affairs	451 female veterans, 217 in RT group, 234 in non-RT group<br><br>At baseline, RT clients had significantly lower total monthly income (Table 1). RT clients were significantly less likely to have minor children, more likely to be black, and less likely to be of "other" race/ethnicity than NRT clients. No baseline differences were noted for days of homelessness, military rank or type of discharge, VA dis ability ratings, education, days worked in the past 30, age, or number of months spent homeless in their lifetime. On clinical measures, RT clients had significantly higher scores on the SF-12 Mental subscale (representing better mental functioning: p = 0.001) and higher ASI Alcohol (p = 0.001) and Drug scores (p = 0.001) (representing more severe substance abuse problems) (Table 2).	Q1: Psychiatric, Alcohol, and Drug composite scales from the Addiction Severity Index (ASI) [16]; the Symptom Checklist-30 (SCL) [17]; and the 12-Item Short Form Health Survey (SF-12) mental subscale [18]. Posttraumatic Stress Disorder (PTSD) Symptom Checklist (PCL) was used to assess PTSD symptoms<br><br>Q2: N/A	Both groups had significantly decreased SCL scores over time. The RT group had significantly lower scores on average (p < 0.001) and at all time points after baseline. The RT group also had significantly lower PTSD scores on average (p = 0.001), specifically at 3 months (p= 0.01), 6 months (p = 0.004), and 12 months (p =0.03). In addition to the overall PCL score, we divided the scale into three subscales. The RT group had significantly lower Hypervigilance scores on average (p < 0.001), specifically at 3 months (p = 0.002), 6 months (p = 0.001), and 12 months (p = 0.003), as well as lower Intrusive Thoughts scores (p = 0.01), particularly at 9 months (p = 0.009). The interaction between RT and time was not significant (p = 0.16). The RT group did not have significantly different Avoidant Behavior scores on average (p = 0.08). The RT group had significantly higher scores on average for the Mental Health component of the SF-12 (p= 0.003), indicating better functioning, particularly at months 3 (p = 0.03) and 6 (p = 0.008). The RT group had	NA

				However, they did not differ significantly on PTSD symptoms, SF-12 Physical scores, social support, or total number of traumatic events		significantly lower scores on average for the Psychiatric ASI scale (p = 0.04), specifically at 3 months (p = 0.005). The RT group had significantly higher scores on average on the ASI Alcohol scale (p = 0.03), controlling for baseline, particularly at 3 months (p = 0.01). When looking at the days of alcohol use, we found that both groups had significantly decreasing days of use over time; however, the RT group had significantly higher use on average (p = 0.03), controlling for baseline, but no particular time point was significantly different. The RT group also had significantly higher scores on average for the ASI Drug scale (p < 0.001), controlling for baseline, particularly at 3 months (p = 0.01) and 6 months (p = 0.001). Both groups had significantly decreasing days of drug use over time; however, the RT group did not have a significantly different change in use (p= 0.07), controlling for baseline.	

Hernandez-Ruiz et al. 2005 USA<br><br>Non-randomised trial	The purpose of this study was to explore the effect of a music therapy procedure (music listening paired with progressive muscle relaxation) on the reduction of anxiety and improvement of sleep patterns in abused women in shelters.	Participants in the control group were instructed to "lie down quietly for 20 minutes" without listening to the music / PMR script	Domestic violence shelter	Twenty-eight women from an upper Midwestern city, mean age 35.36 years, were recruited from two shelters for battered women. They had an average of 2 children (range = 0 to 5), and two of them were pregnant. Most were caring for at least one child in the shelter. The women had been an average of 7.94 years in the last abusive relationship, and the abuse had lasted an average of 4.23 years (range from one day to 34 years). From the total sample, 26 participants reported verbal abuse and 23 also reported physical abuse. The women were referred to the study by the shelter staff if they had been in the shelter for at least 2 days and no more than 1 week.	Q1: To assess anxiety levels, the State Trait Anxiety Inventory was used (STAI)<br><br>Q2: N/A	The first question of this study concerned the effect of a music therapy intervention (music/PMR) on the anxiety level (as measured by the STAI) of women in shelters. Mean scores and standard deviations for each group are shown in Table 1. A Factorial Re-peated Measures ANOVA was completed to examine anxiety data for the two conditions on the first day of treatment (Day 3 of the study). Both a main effect change of anxiety level, $F(1, 26) = 39.04$, $p < .001$, and an interaction by condition, $F(1, 26) = 6.05$, $p = .021$, were found (the latter is graphed in Figure 1). A second Factorial Re-peated Measures ANOVA was conducted to examine the STAI data on both conditions for the second day of treatment (Day 4 of the study). Again, both a main effect change of anxiety level, $F(1, 26) = 17.68$, $p < .001$, and an interaction by condition, $F(1, 26) = 15.73$, $p = .001$, were found (this interaction is graphed in Figure 2).	NA

Herschelet al. 2017 USA<br><br>Cohort study (one group, pre- and post)	Three aims guided the research: (1) to understand the feasibility of delivering PCIT in a community-based domestic violence shelter (e.g., fidelity to the model, treatment barriers and participation), (2) to evaluate the effectiveness of PCIT on key outcome variables including: child behavior, parenting practices, and parental mental health, and (3) to explore barriers to treatment participation contributing to attrition.	No control group. Participants compared with themselves at pre-treatment	Domestic violence shelter	17 parents (of whom 90% female) with children<br><br>The majority of participating parents were biological mothers (n = 17; 81.0%). Parents were primarily single, never married (n = 10; 47.6%), high-school educated (n = 5; 23.8%) and currently unemployed (n = 10; 47.6%). The majority of parents reported experiencing past abuse (n = 16; 76.2%) as well as multiple traumatic or stressful life events. On average, parents reported experiencing 12 traumatic or stressful life events (M = 12.38, SD = 6.92). Please see Table 2 for demographic characteristics of all parent participants	Q1: Parental mental health: The Symptom Checklist-90-R (SCL-90-R) is a widely used, 90-item self-report symptom inventory that measures a broad range of psychological problems<br><br>Q2: The Barriers to Treatment Participation Scale (BTPS) is a 58-item self-report instrument which assesses consumers' perceived barriers to treatment participation. The Therapy Attitude Inventory (TAI; Brestan, Jenifer, Rayfield, & Eyberg, 1999) is a 10-item self-report instrument that assesses parental satisfaction with treatment at the completion of the intervention	Parental self-report of their mental health needs indicated a reduction in symptoms. On average, treatment completers reported 31.4 behavioral health symptoms at pretreatment and reported half as many symptoms (i.e., M = 16.8) at post-treatment. Significant gains were noted with regard to parent scores on the Global Severity Index, t (5) = 3.02, p = 0.03, indicating an overall reduction of behavioral health symptoms, as well as on the Positive Symptom Total scores (i.e., number of positive mental health symptoms reported) following treatment completion, t (7) = 3.38, p = 0.02.	Parental self-report of barriers to treatment (BTPS) indicated the presence of numerous barriers for treatment completers (M = 20.86, SD = 1.77) and non-completers (M = 31.75, SD = 11.70). However, treatment non-completers reported not only the presence of more barriers, but a greater impact of these stressors and obstacles at mid-treatment than treatment completers (t(9)= 2.52, p = 0.03). Notably, barriers remained high at post-treatment for completers as well (M = 19.67, SD = 3.78). All reported high satisfaction at mid-treatment. For treatment completers, treatment satisfaction remained high at post-treatment (M = 46.71, SD = 4.42).

Johnson & Zlotnick 2006 USA<br><br>Cohort study (one group, pre- and post)	The purpose of the present study was to evaluate, in an open trial, the feasibility and initial efficacy of a new cognitive–behavioral therapy designed specifically for battered women with PTSD or subthreshold PTSD living in a shelter (HOPE)	No control group. Participants compared with themselves at pre-treatment (check)	Domestic violence shelters	Of the 18 female participants, 15 met full criteria for domestic violence related PTSD, seven for major depression, seven for a lifetime substance use disorder, and 13 for another anxiety disorder. Average duration of PTSD symptoms was 25.6months; average length of shelter stay was 20 days; and four were on psychotropic medications. Average age was 32 years, and 17 were living with or married to their abuser. On the CTS-2, all women reported psychological and physical abuse, 16 reported abuse with injury, and 10 reported sexual abuse the month prior to shelter. Nine participants were White, seven were African American, two were Hispanic, and two were of another race; 14 women had children, six women were currently employed, and 14 women had a high-school education.	Q1: One-week-symptom severity and PTSD diagnostic criteria was assessed with the Clinician Administered PTSD Scale, and the Beck Depression Inventory was used to assess depression severity.<br><br>Q2: The Client Satisfaction Questionnaire assessed participants' overall satisfaction with HOPE.	Univariate tests found significant effects for PTSD severity and depression (see table 1)	Completers' overall satisfaction with HOPE, as measured by the CSQ's 4-point rating scale, was 3.56 (SD 0.60).

Johnson et al. 2011 USA<br><br>RCT	This study was designed to explore the acceptability, feasibility, and initial efficacy<br>of a new shelter-based treatment for victims of intimate partner violence (HOPE).	All participants received Standard Shelter Services (SSS). SSS included case management, a supportive milieu environment, and attendance of educational groups offered<br>through the shelter (i.e., parenting & support groups). No therapy was offered through SSS.	Domestic violence shelters	70 women: 35 control group, 35 experimental group<br><br>Average age 32.5, 43% White, 50% African American, 4% Latina, 7% other, 90% have children.	Q1: PTSD: measured using the CAPS, a structured interview with established reliability and<br>validity to assess for IPV-related PTSD diagnosis and past-week symptom severity<br>Depression: measured using the Beck Depression Inventory to assess severity of depression symptoms over the past week. The BDI is a 21-item self-report measure of characteristic attitudes and symptoms of depression with established<br>reliability and validity and had excellent internal consistency in the current study (a = .89)<br><br>Q2: Treatment Credibility: The perceived credibility of HOPE was rated on 4-item measure frequently employed in treatment research. Items assessing how logical they found HOPE, how successful they believed HOPE will be in addressing their PTSD and other problems, and their confidence in recommending HOPE were rated on a 9-point scale ranging from 0 to 8.	PTSD: No significant treatment effect was found ($p > .05$). When looking at PTSD symptoms by factors (i.e., re-experiencing, effortful avoidance, emotional numbing, and arousal), a significant treatment effect was found for emotional numbing symptoms, $t(67) = -2.046$, $p <.05$, in which participants in HOPE experienced less severe emotional numbing symptoms over follow-up than did participants in the control group. No significant treatment effects were found for re-experiencing, effortful avoidance, or arousal symptoms (all p's > .05). Chi-Square analyses evaluating PTSD diagnostic status in the MA sample were only significant at 3-months PS, x2 (1) = 4.69, $p <.05$, in which participants in HOPE (15.8%)<br>were significantly less likely to meet criteria for PTSD relative to controls (45.5%), OR =4.44, 95% CIs [1.08–18.22], RR = 2.88, 95% CIs [.95–8.68]. The reliable change index as outlined by Jacobson and Truax (1991) was calculated for CAPS scores at 1-week PS for both the ITT and MA samples. Using a 95% confidence interval, a change of more	Average credibility rating (n = 29) on an 8-point scale was 6.78 (SD = 1.42). Average satisfaction ratings on the 4-point scale of the CSQ (Attkisson & Zwick, 1983) was 3.4 at 1-<br>week PS (n = 32), 3.5 at 3-month PS (n = 31), and 3.4 at 6-month PS (n = 30).

					Client Satisfaction: A 14-item version of the Client Satisfaction Questionnaire (CSQ; Attkisson & Zwick, 1982) was used to assess participant overall satisfaction with HOPE	than 21 points on the CAPS is required to indicate reliable change in the ITT sample. Using this criterion, 63.2% of participants randomized to HOPE achieved reliable change. For the MA sample, a change of 22 points on the CAPS is required to indicate reliable change. Of the MA participants, 64.7% achieved reliable change. Depression: HLM analyses with the BDI for the ITT sample yielded a significant effect of time, in which participants reported fewer depressive symptoms over the course of the follow-up, (67) = 117.71, $p < .0001$. A significant treatment effect was also found, $t(67) = -3.13$, $p < .01$, in which participants in HOPE reported fewer depression symptoms over follow-up relative to participants in the control group (see Table 2). Analyses with the MA sample were similar in that a significant treatment effect was also found, $t(49) = -2.510$, $p < .05$, in which MA participants reported fewer depression symptoms over follow-up relative to participants in the control condition.	

Johnson et al. 2016 USA<br><br>RCT	To evaluate the feasibility, acceptability, and initial efficacy of an expanded 16-session version of HOPE as an adjunct to standard shelter services with those exposed to IPV who sought shelter. Primary outcomes were PTSD severity and re-abuse severity.	All participants received Standard Shelter Services (SSS). SSS included case management, a supportive milieu environment, and attendance of educational groups offered through the shelter (i.e., parenting & support groups). No therapy was offered through SSS.	Domestic violence shelters and community	60 women recruited from homeless shelters. 30 control group, 30 experimental group<br><br>Average age 33.25, 56.7% African American, 43.3% Caucasian, 6.7% Hispanic, 90% have children.	Q1: PTSD: measured using the CAPS, a structured interview with established reliability and validity to assess for IPV-related PTSD diagnosis and past-week symptom severity Depression: measured using the Beck Depression Inventory to assess severity of depression symptoms over the past week. The BDI is a 21-item self-report measure of characteristic attitudes and symptoms of depression with established reliability and validity<br><br>Q2: Client Satisfaction: A 14-item version of the Client Satisfaction Questionnaire (CSQ; Attkisson & Zwick, 1982) was used to assess participant overall satisfaction with HOPE	Latent growth curve analysis yielded significant treatment effects for CAPS scores across time points (ß= -.007, p = .021). Chi-square analyses evaluating PTSD diagnostic status demonstrated a significant effect at 6-months PT x2(1, N = 48) = 6.10, p= .014, in which participants in HOPE + SSSs (18.5%) were significantly less likely to meet criteria for PTSD/subthreshold PTSD than SSSs participants (52.4%), OR = 0.21,95% confidence interval (CI) [0.06, 0.75], RR = 2.20, 95% CI [1.03, 4.72]. No differences, however, were identified at PT, (1, N = 51) = 0.16, p = .690, or 3-months PT, x2 (1, N =50) = 1.09, p = .301. The reliable change index as outlined by Jacobson and Truax (1991) was calculated for CAPS scores at 1-week, and 3- and 6-months PT. Using a 95% CI, a change of more than 26 points on the CAPS was required to indicate reliable change at 1-week PT, 21 at 3-months PT, and 26 6-months PT. Using these criteria, 76.9% of participants randomized to HOPE + SSSs achieved reliable change at 1-week PT, 85.2% at 3-months PT,	Average satisfaction ratings for those who received HOPE + SSSs were 3.68 (SD = 0.36) at 1-week PT, 3.66 (SD = 0.47) at 3-months PT, and 3.62 (SD = 0.43) at 6-months PT on the 4-point scale of the CSQ (Attkisson & Zwick, 1982).

						and 81.5% at 6-months PT. Using the same criteria, 66.7% of participants randomized to SSSs achieved reliable change at 1-week PT, 68.0% at 3-months PT, and 76.2% at 6-months PT.<br><br>Latent growth curve analyses yielded significant treatment effects for BDI scores (ß= -.006, p = .052).	

Johnson et al. 2020 USA<br><br>RCT	Following on from the previous small-scale RCTs by this research team investigating HOPE, the current study evaluated the efficacy of the expanded version of HOPE (Johnson et al., 2016) relative to a time- and attention-matched control condition, over a longer period (i.e., a 12-month follow-up period), and within six diverse shelter settings rather than only one shelter. Our attention-matched control condition was present-centered therapy (PCT), a problem-focused, supportive therapy that has been frequently used as an attention-matched control condition in clinical trials of PTSD. Primary outcomes were PTSD severity and re-abuse severity	PCT+: PCT was designed to control for the non-specific factors of therapy (e.g., therapeutic relationship) and mirror traditional supportive therapy. The critical difference between HOPE and PCT is the focus on trauma. PCT does not include disclosure, discussion, or exposure of individual traumatic events. Further, PCT does not include any of the hypothesized active ingredients of HOPE (e.g., cognitive-restructuring, acquisition of new behavioral skills, including skills that enhance empowerment). The PCT manual used in prior research was adapted for our target population to include safety-planning strategies when participants brought up safety concerns to their therapist (i.e., PCT+).	Domestic violence shelters and community	172 women<br><br>The majority identified as either White (46.5%) or Black/African American (44.2%) and ranged in age from 19 to 59 (M = 35.13, SD = 9.12). For sample demographics by treatment group, see Table 1. The women were sampled from a total of six shelters, with the majority of women recruited from the four larger shelters (i.e., two urban [n = 95] and two rural [n = 57] shelters) and the remaining women recruited from two smaller, rural shelters (n = 20).	Q1: PTSD: measured using the CAPS, a structured interview with established reliability and validity to assess for IPV-related PTSD diagnosis and past-week symptom severity Outcomes Depression Severity.: The Center for Epidemiologic Studies Depression Scale (CES-D; Radloff, 1977) was used to assess severity of depression symptoms<br><br>Q2: Client Satisfaction: A 14-item version of the Client Satisfaction Questionnaire (CSQ; Attkisson & Zwick, 1982) was used to assess participant overall satisfaction with HOPE / PCT	Both treatment groups improved significantly (p < .01) in all outcomes from baseline to each subsequent assessment. However, no significant group differences were found at any occasion (all d-values within ± 0.32) or in changes from baseline to any subsequent occasion (all d-values within ± 0.30) due to relatively small effect sizes. The percentage of HOPE participants who achieved clinically significant change at PT was as follows: 83.1% (n = 54) for PTSD symptom severity, 50.8% (n = 32) for depression severity, 47.7% (n = 31) for posttraumatic cognitions. The percentage of PCT+ participants who achieved clinically significant change at PT was as follows: 76.6% (n = 59) for PTSD symptom severity, 54.8% (n = 40) for depression severity, 25.0% (n = 19) for empowerment, 39.5% (n = 30) for posttraumatic cognitions. No statistically significant difference in rate of reliable improvement by treatment condition was found (all p's > .05).	Average ratings for both HOPE (M = 3.67, SD = 0.40) and PCT+ (M = 3.60, SD = 0.52) were high, reflecting overall satisfaction as good to excellent for both treatments. No significant differences were found in HOPE and PCT+ satisfaction ratings, t(140) = 0.94, p = .351.

Jourilees et al. 2009 USA<br><br>RCT	This study was a randomized clinical trial of Project Support, an intervention designed to reduce conduct problems among children exposed to intimate partner violence. There were four primary objectives. First, we examined effects of Project Support on child conduct problems. Second, we examined effects of Project Support on specific aspects of mothers' parenting. Third, we evaluated effects of Project Support on mothers' psychiatric symptoms in this larger sample. Fourth, we considered whether Project Support works as theorized. Specifically, we evaluated whether the expected changes in mothers' parenting and psychiatric symptoms explained effects of Project Support on child conduct problems.	Project staff attempted to contact families in the comparison condition monthly, either in person or by telephone. These monthly contacts were structured so that these families could receive instrumental and emotional support services similar to those provided to Project Support families. In addition, no restrictions were placed on comparison families' receipt of services from other sources; indeed, we encouraged them to make use of community resources. During the 8-month period following shelter departure, families assigned to the comparison condition averaged 3.7 (SD " 2.66, range " 0 to 9) contacts with project staff in which a safety issue was addressed, emotional support was provided, a referral was requested or offered, some form of instru-mental support was provided, or the family received some	Homes of women who have recently exited domestic violence shelters	66 women with children S	Q1: Mothers' psychiatric symptoms. Mothers reported on the SCL-90-R (Derogatis, Rickels, & Rock, 1976) the level of distress caused by psychiatric symptoms (0 " not at all to 4 " extremely)in the previous week, including anxiety, depression, and somatic complaints. The Global Severity Index of the SCL-90-R was used for analyses.<br><br>Q2: N/A	Mothers' psychiatric symptoms. At the multivariate level, maternal psychiatric symptoms decreased during the intervention period in the Project Support group, and in the comparison group, and the rate of decrease did not differ across the groups. During the follow-up period, psychiatric symptoms continued to decrease in the Project Support group, but not the comparison group, however, the slopes did not differ across the groups,	NA

		combination of support services.					

Kahan et al. 2019 Canada<br><br>Qualitative study with thematic analysis of data	This qualitative study examined the acceptability of the Peer Education and Connection through Empowerment (PEACE) intervention to a vulnerable population of homeless female youth with experiences of domestic violence (MH outcomes of the programme assessed by Bani-Fatemi et al.), and key enablers of successful implementation and engagement.	No control group	Community resource centre for homeless youth with on-site crisis shelter	23 homeless women. The sole direct service provider and program manager were interviewed (n = 2), in addition to one youth worker, one transitional housing team lead and three peer mentors.<br><br>Service user participants were between 19 and 24 years of age. Most were born in Canada, and included Caucasian, Black, South Asian and participants of mixed ethnic background	Q1: NA (measured in Bani Fatemi et al.)<br><br>Q2: Trained research staff conducted 12 semi-structured individual interviews with service users, four semi-structured interviews with frontline service providers and managers and three interviews with peer mentors. The semi-structured interview guides evolved throughout the data collection process, as preliminary findings informed subsequent revisions to better capture interviewees' experiences (Patton, 2015). The interviews lasted between 30 and 90 min and focused on stakeholders' perspectives on program acceptability and key enablers of successful implementation. Service user participants also offered their perspectives on helpful and unhelpful program components, impact on health and well-being and opportunities for program improvement.	NA	Acceptability themes:<br>- Inviting space and limiting barriers to access: The text reminders were seen as particularly helpful by young service users. Providing a safe and inviting space was also seen as reducing barriers to participation and engagement. There was access to a lounge space, and healthy food and beverage options. While the space was seen as aesthetically pleasing and inviting, participants also found the group to have an inviting and respectful atmosphere. Some service users noted that they felt more comfortable talking freely in a mainly female-identified group having shared experiences. Staff and fellow service users were described as non-judgmental, supportive and encouraging.<br>- Co-production, empowerment and choice: Both service users and providers highlighted co-production, empowerment and choice as key components of the intervention. To increase relevance and promote participant empowerment and choice, the intervention was developed with service user input.

					Service provider participants and peer mentors discussed their perspectives and experiences on successes and challenges implementing the program, and engaging and supporting service users.		Participant input was central in framing group norms, developing group educational content and choosing recreational activities and outings. Psychoeducation and group discussion about gender-based violence was viewed as helpful for developing insight into healthy and unhealthy gendered interactions. Such insights ranged from an awareness about violent or abusive situations to assertiveness within intimate relationships. Service user participants also began to challenge previously held distorted beliefs of self-blame related to trauma and abuse. Based on service user requests, psychoeducational sessions were staggered between weeks of recreational activities and outings, which service users viewed as helpful for social cohesion and for giving them a 'break' from difficult material. -Maintaining safety and enhancing learning: Disclosures of trauma occurred spontaneously during open discussion of curriculum content as an unanticipated component of the intervention and appeared to have mixed effects. Participants who

							self-disclosed their own trauma history revealed a generally positive experience, including feelings of validation in the recognition of shared experiences, as well as cognitive and affective processing of trauma. On the other hand, some service users described how other participants' disclosures or certain group content caused distress. Despite overall positive feedback by most participants, one service user chose to leave the program due to difficulty tolerating group discussions and feeling unprotected from triggering material.<br>- Making the most of lived experiences: A key ingredient of the intervention was peer mentorship, fostered by building group cohesion and by group facilitation from trained peer mentors. Group cohesion was demonstrated through the sharing and appreciation of common experiences, as well as the instillation of hope, and altruism from being able to help others. In response to concerns that trauma disclosures were causing distress in some participants, staff in later groups cautioned participants against sharing specific details of

							their trauma experiences if choosing to self-disclose. Overall, service user experiences with peer mentors were positive. A number of service users described seeing peer mentors as more approachable or accessible because they were not health or social service professionals.

Kim & Kim 2001 South Korea<br><br>Cohort (2 groups pre and post; non-randomised trial)	To develop an appropriate group intervention model for battered women in Korea and to test its effectiveness. The group intervention was expected to decrease depression and anxiety, and to increase self-esteem of battered women staying in shelters.	The control group were selected from a separate shelter and received shelter services as usual, which did not include any group or individual counselling / therapy	Domestic violences shelters	60 women (30 intervention, 30 control).<br><br>The age of the subjects in the experimental group ranged from 23 to 43 years with a mean age of 35.8 years. The age of the subjects in the control group ranged from 28 to 52 years with a mean of 36.8 years. The majority of the subjects (N514, 87.5%) in experimental group and 17 subjects (100%) in the control group had not received further education after high school. No p-values were reported to check for differences in demographic characteristics between the two groups	Q1: The Center for Epidemiological Studies Depression Scale (CES-D) measures symptoms of depression in the general population. Spielberger's State-Trait Anxiety Inventory (STAI) was used to measure current and general levels of anxiety.<br><br>Q2: N/A	Scores on state anxiety were significantly lower in the post-test than in the pretest in both groups (t=2.50, p=.025 ; t=3.52, p=.00, respectively).<br>The trait anxiety scores were also significantly lower in the post-test than in the pretest in the experimental group (t=4.30, p=.00). However, there was no significant change in the control group on trait anxiety.<br>The change in level of trait anxiety over time in the experimental group was significantly different from those of women in the control group (t=-3.20, p=.00).<br>Changes in the level of state anxiety in the experimental group were not significantly different from that of the control group.<br>A paired t test showed no statistically significant increase in self-esteem scores (indicating higher self-esteem) between the pre-test and post-test in either the experimental or control group.<br>A statistically significant difference in the level of depression was found between the pre-test and post-test scores for the experimental group (t=3.134, p=.007). The score changes for the level of depression between the pre-test and	NA

						post-test in the experimental and the control groups were-13.31 and -5.76 respectively. However, the change in level of depression over time in the experimental group was not significantly different from those of women in the control group.	
Lako et al. 2018 Netherlands<br><br>RCT	To examine the effectiveness of critical time intervention (CTI)—an evidence-based intervention—for abused women transitioning from women's shelters to community living.	Women in the control condition received care-as-usual. All organizations provided services after discharge, except for one which referred women returning to their (ex-)-partner to other services. Most organizations provided support during regular meetings (range of average intensity: 1–3 h per week). The average duration of these services varied widely between organizations	Homes of formerly homeless women	136 women (70 experimental group, 66 control group)<br><br>The control group contained more first-generation migrants and more married women. In the experimental group, more women experienced sexual violence. Outcome measures showed some differences between groups at baseline: women assigned to CTI reported fewer symptoms of depression and psychological distress but more	Q1: All outcomes were assessed at T0 and T9 with the exception of the outcome re-abuse, which was measured at T3, T6, and T9. The sum score of the 20-item Center for Epidemiological Studies Depression scale was used to measure depressive symptoms. PTSD symptoms were measured by the sum score of the 15-item Impact of Event Scale. The Global Severity Index, an average score of the 53-item Brief Symptom Inventory was used to assess psychological distress.	There was no significant between-group difference in QoL during follow-up (Table 3). For the secondary outcomes, women in the experimental group experienced significantly less symptoms of PTSD during follow-up (adjusted mean difference - 7.27, 95% CI - 14.31 to - 0.22, p = 0.04). No between-group differences were found for the other secondary outcomes	NA

		(range: 13–52 weeks). All organizations employed a strengths=based approach.		symptoms of PTSD than women assigned to care-as-usual	Q2: N/A		

Mallory et al. 2022 USA<br><br>RCT	To compare treatment responses between sexual minority (SM) youth and heterosexual youth participating in a comprehensive housing intervention for young mothers experiencing homelessness.	Control group: (1) housing only and (2) SAU – referral sheet including all services available in the community	Not stated	240 young mothers (18-24 years) experiencing homelessness<br><br>Participants were primarily heterosexual (78%) and Black/African American (86%), average age of 21.6 years old (SD = 1.80)	Q1: 1.Substance use measured using the Form-90<br>2.Depressive symptoms measured by Beck Depression Inventory II<br><br>Q2: N/A	Heterosexual mothers receiving SAU or Housing + SS experienced a decline in depression symptoms, while only SM mothers in the housing + SS conditions showed a significant decline in depression symptoms. Heterosexual and SM mothers who received Housing + SS and heterosexual mothers receiving SAU experienced a decline in depression symptoms and maintained these during the twelve months of the study at similar rates.<br>However, SM mothers who received SAU did not have any changes in their depression symptoms during the twelve months.	NA
Marin et al. 2021 Spain<br><br>Cohort study - one group	This study aims to examine the feasibility of the Unified Protocol for Transdiagnostic Treatment of Emotional Disorders among homeless women, in relation to its quantitative effects on attendance, **satisfaction**, usefulness, emotional state, and group cohesion.	No control group. Participants were compared with themselves at pre-treatment	Four homeless shelters	Their mean age was 49.57 years old (SD 10.78), and they were mainly of Spanish nationality (57.4%). Of the participants, 44.4% were single, 37.0% were separated/divorced, and 16.7% were married/in a stable relationship. Although 70.4% of the women had children, none of them were living with their children at the time of the interview. The mean time spent being homeless by the women interviewed was 89.16 months (SD 115.18 months).	Q1: NA<br><br>Q2: The Participant Feedback Session Survey was developed ad hoc and was completed anonymously after each session. It consisted of four items that measured the following: (a) satisfaction with the session: participants answered the question "How much did you like this session?" using a 10-point Likert scale; (b) perceived usefulness of the session: participants answered the question "How useful do you find the information given in this session?" using a 10-point Likert scale; (c) mood and emotional state: participants answered the question	NA	Overall, participants reported high total levels of satisfaction (M=8.97, SD=1.45), perceived usefulness (M=9.10, SD=1.42). The results of the Mann–Whitney U tests revealed significant increases in participant satisfaction (U=417.00, p=.001), and perceived usefulness (U=468.50, p=.003) between sessions 1 and 12.

					"How do you feel in this moment?" using a visual analog scale with responses ranging from 0 to 7; (d) group cohesion: participants answered the question "How did you feel with the group?" using a visual analog scale with responses ranging from 0 to 7.		

Noh et al. 2018 South Korea<br><br>Non-synchronised cohort (2 groups pre- and post-, non-randomised)	This study developed a resilience enhancement programme considering the context of Korean female runaway youths and tested the effects of the programme on resilience, depression, anxiety, and problem drinking among them.	The control participants received service as usual through youth shelters. The provided service included a place to reside, basic subsistence items, such as food and hygiene supplies, and advice for daily living and counselling provided by shelter staff.	Youth homeless shelters	32 homeless female youth (16 in experimental group, 16 in control group)<br><br>Participant age range was 12–21 years and the mean age was 16.69 years (SD=2.56). Most participants were current middle or high school students (62.5%) and were of low reported family socioeconomic status (75.0%). The mean family function score was 1.97 (S=2.76), indicating severely dysfunctional family life.	Q1: Depression = The Beck Depression Inventory-II (BDI-II), translated into Korean was used.<br>Anxiety = The Beck Anxiety Inventory (BAI) translated into Korean<br>Problem drinking = The Alcohol Use Disorders Identification Test Alcohol Consumption Questions (AUDIT-C), based on questions 1–3 from the AUDIT developed by the World Health Organisation<br><br>Q2: N/A	NA	Significant group-by-time interaction effects were seen for resilience between pre-test and both post-test (beta=12.42, p=0.002) and 1-month follow-up (beta=12.72, p=0.007). A significant increase in resilience over the study period occurred for experimental participants when compared to control participants, whereas the control group showed decreases in resilience over all time periods.<br>In terms of depression, a significant group-by-time interaction was seen between pre-test and post-test (beta=-5.33, p=0.037), but not between pre-test and one month follow-up (beta= 4.48, p=0.120). In contrast, there was a significant time effect between pre-test and one-month follow-up (beta=-3.33, p=0.030). That is, a significant decrease in depression for experimental participants occurred during the one-month intervention period, but not during the overall study period, because decreases in depression over the study period occurred for both the experimental and control participants.<br>In terms of anxiety, a significant group-by-time interaction was seen between pre-test and one-month follow-up (beta=8.00, p=0.022). The	NA

							average level of anxiety decreased consistently over the study period in the experimental group compared to the control group, whereas an increase in anxiety occurred for control participants at one-month follow-up. Significant group-by time interaction effects were seen for problem drinking between pre-test and both post-test (beta=3.58, p<0.001) and one-month follow-up (beta=-0.63, p=0.038). The average level of problem drinking decreased consistently across all time periods in the experimental group, the control group had average levels of problem drinking at post-test (M=2.69, S=2.95) and one-month follow-up (M=2.54, S=3.18) that were higher than that at pre-test (M=2.50, S=2.92).	

Nyamathi et al. 1998 USA<br><br>RCT	This research examined the impact of including a supportive person on the outcomes of two culturally sensitive AIDS education programs, an education only (traditional) program and a program combining education with self-esteem and coping enhancement (specialized). The outcomes were risk behaviors, cognitive factors, and psychological functioning.	Women in the traditional intervention program received in small group format a 2-hour culturally sensitive AIDS education program by a nurse and outreach worker of their ethnicity with other women only (Traditional Group 1) or with other women and their SPs (Traditional Group 2) at Time 1. Over the subsequent seven weeks, these women alone or with their SPs, continued to receive a 45-minute session each week wherein condoms and bottles of bleach were dispensed and reinforcement of AIDS information was provided. Reinforcement of information at 6- and 12-month follow-up was also provided lasting 30 minutes each session. The program delivered basic AIDS education consisting of content related to AIDS etiology, symptoms, modes of transmission, methods of protection, pros and cons of HIV testing and meaning of HIV results.	Community centres in Los Angeles	241 homeless women (plus their partners)<br><br>Participants with baseline and 12-month follow-up data were 169 predominantly African American and Latina impoverished women (Table 1). The 169 women ranged in age from 21 to 63, with a mean age of 35 years (SD = 7). The women were almost exclusively African American (91%), Protestant (75 %), and not married (81%). Years of education ranged from 3 to 17 years, with a mean of 12 (SD = 1.8). The vast majority of the women were unemployed and had children (90%).	Q1: Mental health was measured by the MHI-5 (Mental Health Index-5) - good validity for detecting MH disorders such as depression and anxiety Depression was measured by the Center for Epidemiological Studies Depression (CES-D) Scale Non-injecting drug use was measured as part of a four item Risk-taking behavior scale that assessed the degree to which subjects engaged in behaviors that put them at risk for AIDS (does not appear to be validated and appears to have been created by authors for the purposes of this study).<br><br>Q2: N/A	NA	Psychological well-being scores were noted to improve over time for women in both programs, and for those with and without participating partners. For the sample as a whole, mean psychological well-being scores increased from 49.9 at baseline to 62.9 at 12 months. Profiles for depression were similar, with the sample improving from a mean of 28.5 at baseline to 21.6 at 12 months, but no differential effects were noted with respect to type of program or having a participating partner for either psychological wellbeing or depression.<br>Only 10 women reported use of injection drugs at baseline, making this behavior too rare for analytic purposes. It is noteworthy, however, that only two women reported injection drug use at 6- and 12-month follow-up. In contrast, two-thirds (66%) of women in the study reported use of non-injection drugs at baseline. Significant reductions in non-injection drug use were evident at 6-month follow-up (27%), followed by a small increase at 12 months (32%). However, a significant interaction with program type was found, with women in the specialized program improving more between baseline and 6 months and then maintaining that	NA

							improvement as compared to traditional program women. No significant interaction between time and partner presence was identified.	

Nyamathi et al 2017. USA<br><br>RCT	The primary purpose of this study was to determine the effect of a DBT-CM intervention program versus a health promotion (HP) program on drug use abstinence among homeless female parolees/probationers at six-month follow-up. As secondary objectives, we examined the effect of the intervention on abstinence from alcohol use and combined drug/alcohol use	HP program (comparator)—For participants assigned to the HP program, a dedicated nurse and two CHWs were trained to deliver a program focused on common chronic diseases that homeless women face and health promotion activities for these chronic diseases. Similar to the DBT-CM program, the women met in small groups of 5–7 at a time to discuss a particular chronic disease as well as in one-on-one sessions with the nurse or CHW to discuss more personalized strategies. The six HP sessions, conducted weekly, were focused on: (a) diabetes, (b) heart disease, (c) sexually transmitted infections, including HIV, (d) parenting skills, (e) community and family reintegration, and (f) other topics. The program was delivered over 12 weeks; there was no ongoing meeting of the participants in relation to referrals and support.	Community-based partner sites	130 homeless female ex-offenders (65 control, 65 experimental group)<br><br>Most participants were Black or Latina and the majority were unemployed. In total, 70% of the participants were on probation at the time of enrolment. Participants reported moderate levels of social support and coping behavior.	Q1: Alcohol and drug use was self-reported using the Texas Christian University Drug History (TCU) form II. A 5-panel FDA-approved urine test cup (Phamatech, Inc.) was used at baseline and 6-month follow up. The test cup screened for metabolites of amphetamines, cocaine, methamphetamines, 3,4-methylenedioxy-methamphetamine (MDMA), opiates, and marijuana.<br><br>Q2: N/A	NA	At the six-month follow-up visit, 65.5% (38/58) of DBT-CM participants and 48.3% (28/58) of HP participants were abstinent for drug use, based on urinalysis confirmation of self- report (Table 2). Drug abstinence increased at six-month follow-up in both groups compared to the baseline. However, the magnitude of the increase in drug use abstinence was greater in the DBT-CM group compared to the HP group (i.e., the interaction term was significant; OR= 2.60; 95% CI [1.04, 6.53]; p = .04). Similarly, participants in the DBT-CM group were more likely to become or remain alcohol-abstinent during the study period (OR = 3.12; 95% CI [1.24, 7.85]; p = .02); the HP group did not change. The differences in increased odds of substance abstinence (abstinent for both drugs and alcohol) was not significant (i.e., the interaction term was nonsignificant; OR = 2.39; 95% CI [0.92, 6.23]; p = .07). When missing outcome data at six months were imputed by carrying the baseline data forward, the DBT-CM informed program had greater effect on alcohol abstinence than the HP program (p = .02), while the differences between the two programs for substance abstinence (p < .11) did not reach statistical significance.	NA

							In multiple imputation analysis, the difference effect between DBT-CM and HP groups did not reach statistically significance for any of the three outcomes. Table 3 shows the final multivariable logistic regression model, including all variables associated with drug use abstinence at six months (p < .05). DBT-CM program remained a positive predictor of drug use abstinence at six months (aOR = 3.15; 95% CI [1.30, 7.69]; p= .01).	
O'Campo et al 2023 Canada<br><br>RCT	To examine the effectiveness of Housing First intervention in a sample of homeless women from five Canadian cities, and explore baseline risk factors that predict social, health and well-being outcomes over a 24-month period.	TAU: access to housing and services through other community programmes.	Homeless shelters	Housing First: N=374; TAU N=279.<br><br>15% born outside of Canada, 3% employed at baseline and 90% received social assistance. Around 40% had minor children and of those 22% were fully or partially financially supporting their children. The most prevalent mental illnesses were depressive episodes, PTSD, and alcohol or substance dependence. Around 40% had moderate-to-high	Q1: 1.Quality of life measured by The Lehman Quality of Life Interview 20<br>2. Community functioning measure by Multnomah Community Ability Scale (MCAS)<br>3.Psychiatric symptoms measured using The Colorado Symptom Index (CSI)<br>4. Self-reported integration with the immediate community measured by The Community Integration Scale (CIS)	NA	Over the 24-month follow-up period, improvements were observed for all the outcomes for both HF and TAU group. Mean total QoLI-20 scores improved 16.0 points (95%CI = 13.6–18.4) and 13.4 points (95%CI = 10.6–16.2) in the HF and TAU groups, respectively, and number of ED visits decreased by approximately 60% in both groups. The mean percentage of days spent stably housed during follow-up for women	

				suicidality, around 40% had PTSD, about 60% reported 5 ACEs, and the majority had ≤ 3 co-morbid medical conditions	5. Past month substance-related problems measured by Global Assessment of Individual Needs Short Screener (GAIN-SS)<br>6. Stability of housing measured by calculating the percentage of days stably housed during past 24 months.<br><br>Q2: N/A		receiving HF was 74.8% (95%CI = 71.7%–77.8%) compared with 37.9% (95%CI = 34.4%–41.3%) in TAU. The HF group also showed significant improvements in psychological community integration by 0.7 points at the 6-month follow-up. Suicidality was a consistent predictor of increased mental health symptoms (beta = 2.85, 95% CI 1.59–4.11, p<0.001), decreased quality of life (beta = -3.99, 95% CI -6.49 to -1.49, p<0.001), decreased community functioning (beta = -1.16, 95% CI -2.10 to -0.22, p = 0.015) and more emergency department visits (rate ratio = 1.44, 95% CI 1.10–1.87, p<0.001) over the study period.	

Q2: N/A

Rodriguez-Moreno et al. 2020 Spain<br><br>Quasi-experimental waitlist controlled pre–post-test design with repeated measures	The purpose of this study is to evaluate the effectiveness of the Unified Protocol for Transdiagnostic Treatment of Emotional Disorders adapted for homeless women (UPHW). Primary outcomes included depression and anxiety.	Waitlist participants received psychological and pharmacological Treatment-As-Usual (TAU): usual shelter services, including individual session with the shelter psychologists, group occupational therapy sessions and employability workshops. Participants assigned to WLC did not immediately receive the intervention for 3 months, after which they received the same treatment as those in the UPHW condition	Homeless shelters in Madrid	81 homeless women (46 intervention group, 35 waitlist control)<br><br>Participants' mean age was 49.45 (SD = 9.76), 47.8% were Spaniards, 40.6% were single, 23.1% had a high education level and 92.8% were unemployed.	Q1: Primary outcome measures were severity of anxiety and depression symptoms, as measured by the Beck Anxiety Inventory (BAI) and Beck Depression Inventory-II (BDI-II) respectively. Secondary outcomes measures included: Anxiety and depression severity and functional impairment, measured with the Overall Anxiety Severity and Impairment Scale (OASIS) and the Overall Depression Severity and Impairment Scale (ODSIS).<br><br>Q2: N/A	NA	Primary outcome measures (BDI and BAI). ANCOVAs analyses on the baseline-corrected post-intervention scores showed a significant group effect for both depression and anxiety Pairwise Bonferroni corrected comparisons indicated that depression and anxiety scores were significantly lower in UPHW than WLC at post intervention. Secondary outcome measures (PANAS, PHI, SF-12 and SSQ): ANCOVAs analyses on the baseline-corrected post-intervention scores showed a significant group effect for negative affect Pairwise Bonferroni corrected comparisons indicated that negative affect was significantly lower in UPHW than in WLC at post intervention. However, ANCOVA analyses did not show significant group effect for positive affect or psychological wellbeing. Follow up effects of the UP adaptation for homeless women: Repeated measure ANOVAs were	NA

							computed to test whether the post changes remain stable over time (i.e., 3-month and 6-month follow up). ANOVAs showed a significant time effect for both depression (Pairwise Bonferroni corrected comparisons indicated that depression and anxiety reductions after the intervention remained in the 3-month follow up (post to 3-month 0.05), however, these differences were no longer present at the 6-month follow up. Regarding negative affect, repeated measure ANOVA also showed a significant time effect, however, pairwise Bonferroni corrected comparisons indicated that negative affect reduction after the intervention vanished in the 3-months and 6-months follow up.	

Rodriguez-Moreno et al. 2022 Spain<br><br>Controlled before-and-after-study	To understand potential mediators and moderators in an adaptation of the Unified Protocol for Homeless Women (UPHW)	Waitlist control (WLC)	Homeless shelters in Madrid	80 women experiencing homelessness and attended at least 6 sessions: 37 in the UPHW and 43 in the WLC<br><br>Intervention group: mean age 49.92 (SD=11.28), 48.6% single, 65% Spanish nationality, time in a homeless situation (in total) 79.93 (SD=99.71); mean depression (BDI score)=20.51 (SD12.38); anxiety (BAI score) = 20.14 (SD=12.91). Control group: mean age 49.49 (SD=9.96), 34.9% single, 56% Spanish nationality, time in a homeless situation (in total) 86.83 (SD=119.01); mean depression (BDI score)=17.98 (SD13.41); anxiety (BAI score) =16.58 (SD=13.41).	Q1: 1.Anxiety and depression symptoms measured by Beck Anxiety Inventory (BAI) and Beck Depression Inventory-II (BDI-II)<br>2. Emotional functioning measured by Positive and Negative Affect Scale (PANAS)<br>3. Integrative wellbeing measured by the Pemberton Happiness Index (PHI)<br>4. Health status was measured by the Short Form Health Survey (SF-12<br>5. Social support was measured by the Social Support Questionnaire (SSQ6)<br>6. Anxiety and depression severity 7. Functional impairment, measured with the Overall Anxiety Severity and Impairment Scale (OASIS) and the Overall Depression Severity and Impairment Scale (ODSIS)<br><br>Q2: N/A	NA	The UPHW group showed significant improvements on anxiety and depression outcomes at post-intervention compared to WLC. Negative affect was the only significant mediator of the relationship between attending the UPHW and reductions in anxiety and depression. Physical functioning was the only significant moderator of the relationship between the UPHW and depression symptoms.	NA

Rodriguez-Moreno et al. 2022 Spain<br><br>Controlled before-and-after-study	To evaluate the effectiveness of the Unified Protocol for Transdiagnostic Treatment of Emotional Disorders adapted for homeless women (UPHW).	Waitlist control (WLC)	Homeless shelters	81 homeless women who: (1) lived outdoors (on streets, public space, or outside); (2) in emergency accommodation; (3) in accommodation for homeless people<br><br>Participants' mean age was 49.45 (SD=9.76), 47.8% were Spaniards, 40.6% were single, 23.1% had a high education level and 92.8% were unemployed. The average age of arrival to a homeless situation was 40 - 45years (SD=14.34), the average total time in a homeless situation 7.24 years (SD=9.10) and the average number of times in a homeless situation was 1.83 times (SD=0.69).	Q1: 1.Anxiety and depression symptoms measured by Beck Anxiety Inventory (BAI) and Beck Depression Inventory-II (BDI-II)<br>2. Emotional functioning measured by Positive and Negative Affect Scale (PANAS)<br>3. Integrative wellbeing measured by the Pemberton Happiness Index (PHI)<br>4. Health status was measured by the Short Form Health Survey (SF-12<br>5. Social support was measured by the Social Support Questionnaire (SSQ6)<br>6. Anxiety and depression severity 7. Functional impairment, measured with the Overall Anxiety Severity and Impairment Scale (OASIS) and the Overall Depression Severity and Impairment Scale (ODSIS)<br><br>Q2: N/A	NA	The UPHW resulted in significant improvement on measures of anxiety (F(1, 66)=4.79, p=.03, η2 p=0.07; 1−β=0.58), depression (F(1, 66)=16.90, p<.001, η2 p=0.20; 1−β=0.98) and negative affect (F(1, 66)=5.18, p=.03, η2 p=0.07; 1−β=0.61) . Improvements in anxiety and depression were maintained over a 3-month follow-up period, but not at 6-month. Negative affect reduction after the intervention was not maintained after the 3-months and 6-months. The percentage of participants in UPHW group achieving functional changes in depression (78.8%) was larger than in WLC (43.8%). The percentage of participants deteriorated after the intervention was significantly larger in WLC (25%) than in UPHW (3%).The inter-session assessment in the UPHW group showed a linear trend reduction for depression and anxiety scores along the 12 sessions.	

Sacks et al. 2004 USA<br><br>Quasi-experimental non-equivalent control group design	The current project, evaluates a homelessness prevention therapeutic community (HP–TC) for addicted mothers and their children in comparison with a standard TC program. The study compares the experimental (E) condition in two HP–TC programs (New Image and Kindred House), with the comparison (C) condition in two standard residential TC programs (Concept 90 and West Chester) in Pennsylvania across a range of outcomes.	Traditional residential TC methods that employed mutual self-help and the peer community to facilitate change. No more detailed information in the study	4x residential drug treatment centres	196 women<br><br>The subjects can be characterized as predominantly minority (80%), average age 33, never married (66%), from broken homes (71%), and with less than a high school education (53%). An average of three children were reported, yet only one child was residing with the mother at baseline.	Q1: The psychological distress outcome domain was a composite of 3 measures: BDI, ASI and SCL-90-R.<br>The substance abuse outcome domain was a composite of measures of Any illegal drug use, frequency of use and number days use (crack/cocaine, marijuana, alcohol intoxication, heroin/opiates), number of different types of illegal drugs used, number of types of alcohol and drug use impacts<br><br>Q2: N/A	NA	Because the standard inclusion of statistical control variables may not have dealt adequately with the many significant differences between E and C subjects at baseline (see Table 3), this article uses an aggregate co variate— the propensity score—to adjust for non-equivalencies between subjects in the E and C groups. Propensity scores help to match subjects, excluding those for whom few or no equivalent subjects exist in the other group. The cases were then divided into three propensity strata of equal size, which showed clearly that two strata lacked adequate numbers of either E or C subjects: Low propensity for the E group (stratum 1), which had only four individuals in E, and High propensity for the E group (stratum 3), which had only six individuals in C. The remainder of this paper examines the Medium propensity group only (stratum 2), which has a satisfactory number of matching cases (21	NA

							clients in E and 28 in C), and which contains one-third of the full study sample. The propensity score model greatly improved the similarity between the E and C groups. The findings reported below focus on the effect size of the experimental treatment, which succinctly compares differential change between two groups. ''Hedges g'' effect sizes (Rosenthal, 1994) were scaled so that positive effect sizes reflect more improvement in the E group than the C group (negative effect sizes reflect more improvement in the C group). The results indicate significantly better outcomes for the E as compared to the C group for the Psychological distress domain ($p<0.05$) but not for the substance abuse domain ($p>0.05$).	

Salem et al. 2017 USA<br><br>RCT	The purpose of this pilot randomized controlled trial (RCT) was to compare the efficacy of a Frailty Intervention (FI) versus a Health Promotion (HP) program among pre-frail / frail homeless women. Outcomes were physical, psychological and overall frailty, drug dependence and alcohol and drug use	The HP program consisted of six group education sessions in which each session lasted up to 60 min and was led by a separate CHW interventionist. These session topics included: (1) general community resources, (2) safety, (3) hypertension, (4) diabetes, (5) arthritis, and (6) cholesterol.	Homeless day centre	32 homeless women (17 HP, 15 FI)<br><br>The mean age of participants was 54.78 (ages 41–72; SD 6.77). The majority was African American (84.4%), fewer were Anglo/White/Caucasian (9.4%), equal numbers were mixed (3.1%) and other Hispanic (3.1%). Approximately half of the sample slept in a shelter (46.9%), followed by those living in unsheltered environments (37.5%).	Q1: Drug dependency, any alcohol use and any drug use were measured by the Texas Christian University Drug History (TCU) form (Knight et al. 2002), which assessed the frequency of alcohol use within the last 3 months. Responses were coded as "1" if individuals responded that they had used any alcohol or drugs (e.g., marijuana, crack, methamphetamine, amphetamine, tranquilizers) in the last 3 months (Knight et al. 2002).<br><br>Q2: N/A	NA	The group by time interaction effect (controlling for covariates in the model) was not significant (at p= .05) for any of the outcomes, indicating that FI and HP programs did not differ significantly in their pattern of change from baseline to 3 months follow-up. However, several of the estimated effect sizes were in the medium-to-large range, suggesting potential impact of the HP program (if results are replicable in a larger sample): d = 0.73 (drug dependency), d = 0.90 (any drug use), respectively and OR 2.52 for any alcohol use.	NA

Samuels et al. 2015 USA<br><br>RCT	The present study examined the effectiveness of Family Critical Time Intervention (FCTI) plus housing vs homeless services as usual (including housing), on maternal mental health for homeless mothers experiencing mental health and substance abuse problems.	Shelter services as usual. Shelter sites typically provided basic on-site services that included, but were not limited to, physical and mental health assessment and treatment; case management; substance abuse screening and rehabilitation; childcare, recreation, and after school programs; parenting, adult education, life skills, and job-readiness programs; and home-finding programs. Although shelter personnel provided many of the on-site services, nonprofit and private agencies were also co-located at some shelters.	Homeless shelters and newly acquired community housing	210 mothers who headed homeless families (97 FCTI, 113 control)<br><br>Most of the homeless mothers (85%) identified as African American, Latino, or other ethnic minority; this percentage contrasted with the primarily (71.3%) Caucasian population of the county as described in Census data. The majority of mothers were in their late 20s and early 30s, living with 3 children (SD = 1.6) younger than 18 years. The average age of the children was 9 years old (SD = 5.00). Nearly two-fifths of the mothers did not have a high school diploma or GED, and most (85%) were currently unemployed. More than 1 out of 5 of the mothers reported that during their childhood, they had been involved in foster care placements.	Q1: Maternal mental health: Maternal mental health symptoms were measured using the Brief Symptom Inventory (BSI; Derogatis, 1993). The BSI, a shortened revised version of the Symptom Checklist-90.<br><br>Q2: N/A	NA	Maternal MH: Time negatively predicted change in symptoms, such that all mothers experienced a decline in mental health problems across the 15-month follow-up period. No significant differences existed in initial mental health and rate of decline between treatment conditions; mothers receiving FCTI experienced similar improvements in symptoms as families in the control condition.	NA

Shores et al. 2014 USA<br><br>Non-randomised trial	In a pilot "proof-of-concept" study, we provided supervised Mental and Physical (MAP) Training (2 sessions per week for 8 weeks) to a group of young mothers in the local community who were recently homeless, most of them having previously suffered from physical and sexual abuse, addiction, and depression. The aims were to determine whether MAP Training improves dependent measures of aerobic fitness (as assessed by maximal rate of oxygen consumed) as well as decreases symptoms of depression and anxiety.	Women living in the same shelter who received shelter services as normal without the MAP intervention	Homeless shelter	14 women (8 MAP, 6 control)<br><br>Demographic characteristics not reported	Q1: Depression symptoms measured by Beck Depression inventory (BDI). Anxiety symptoms measured by Beck Anxiety Inventory (BAI).<br><br>Q2: N/A	NA	After 16 sessions of MAP Training (twice a week for eight weeks), participants expressed significant changes in mental health outcomes. Before training, participants reported higher symptoms of depression consistent with a diagnosis for major depressive disorder (MDD), as assessed with the Beck Depression Inventory (BDI-II; Beck, Steer, & Carbin, 1988). After 8 weeks of MAP Training, mean BDI scores decreased significantly. As shown in Figure 5B, BDI scores after MAP Training were less than half what they were before MAP Training. Before training, the participants also expressed higher symptoms of anxiety as detected with the Beck Anxiety Inventory (Beck, Epstein, & Brown, 1988). Following MAP Training, BAI scores were significantly reduced, suggesting that participants were experiencing fewer symptoms of anxiety. The same mental health outcomes were assessed in a comparison group of	NA

							previously homeless women who live at the center but who did not participate in the MAP Training program (n=6). 6). No change in BDI [F(1,5)=0.14; p=0.72] or BAI [F(1,5)=1.21; p=0.32] scores were found over the course of 8 weeks in women living under similar conditions without participation in MAP Training. With respect to depression, there was a significant interaction between MAP Training and time of assessment, F(1,12)=7.61; p<0.05. As noted, only those who participated in the MAP Training intervention reported decreases in depression symptoms. With respect to anxiety, there was no interaction between the intervention and time between testing periods (pre to post assessment) because both groups (MAP Trained and TAU) reported a decrease in anxiety symptoms over time. This is not surprising because the participants in the center program also experience other changes in their lives that are known to	

							enhance mental health, including other psychological programs and group therapies, as well as medical assistance and nutritious food and the comforts of heat and shelter. Therefore, we are not claiming that 8 week of MAP Training is responsible for all of the positive mental and physical health outcomes, but rather that it synergistically enhances the health outcomes that were observed, especially those related to depression. It is further noted that we have observed similar outcomes in a group of depressed adults and otherwise healthy controls. In these studies, we have also observed a significant decrease on BDI scores after MAP Training, even in the healthy control group (Alderman et al., 2014ab).	

Slesnick & Erdem 2012 USA<br><br>Cohort (one group pre and post)	This study pilot-tested a comprehensive intervention termed Ecologically Based Treatment (EBT) with 15 homeless women and their 2- to 6-year-old children, recruited from a local family shelter. The intervention had three major components. The first component was housing which included 3 months of rental and utility assistance, and these services were not contingent upon women's abstinence from drugs or alcohol. The second and third components included 6 months of case management services and an evidence-based substance abuse treatment (Community Reinforcement Approach; CRA). Primary outcomes were substance abuse, mental health and housing.	No control group. Women were compared with themselves at pre-treatment	Women were recruited from homeless shelters; the intervention took place in part at the homeless shelter and in part at the community once they had been housed	15 homeless mothers<br><br>Mean age 25.2, 66% African American, mean 2.4 children	Q1: Substance use: The self-reported substance use was measured via the Form 90 drug and alcohol interview - a semi-structured instrument. Women also provided urine toxicology samples<br>Mental health: Mental health: The Short-Form-36v2 (SF-36v2) was utilized as a general assessment of health status of the homeless mothers. The current study utilized the SF-36v2 mental health composite score as an overall indicator of mental well-being. -II50) The Beck Depression Inventory-II (BDI was administered to the homeless mothers to assess their depressive symptoms<br><br>Q2: N/A	NA	Substance use: The one-way repeated measures ANOVA revealed a significant main effect of time on percent days of substance use in the prior 90 days. Specifically, mothers had reduced the percent days of substance use at the 3-month assessment (M = 18.9%), compared to baseline (M = 48.8%; p = .013). Substance use started to increase at the 6 month follow up (M = 33.7%), but did not reach significance (p> .05). The analysis was re-run with the log-transformed variable and results suggested no main effect for substance use over time.<br>Mental Health: — Mothers' mental well-being, as assessed by their SF-36v2 Mental Health composite scores, significantly improved over time . It was found that mothers had better mental health at 3 months (M = 43.8) and 6 months (M = 45.5), than at baseline (M = 36.6). Trend analysis on SF-36v2 scores revealed a significant linear trend towards improved mental well-	NA

							being among the mothers. Similarly, mothers reported having fewer depressive symptoms at 3 months (M = 22.9) than at baseline (M = 24.6), and their symptoms continued to decrease at 6 month follow up (M = 17.9). However, reductions in BDI-II scores did not reach statistical significance.	

Slesnick & Erdem 2013 USA<br><br>RCT	To test a systematically-developed housing intervention, combined with ongoing substance abuse treatment and case management, referred to as Ecologically-Based Treatment (EBT), for substance abusing homeless mothers with young children in their care. Primary outcomes were housing and substance abuse.	TAU includes emergency shelter for women and their children up to three weeks at the shelter and linkage to housing and support services in the community. The shelter conducts "rapid re-housing" with the goal for all clients to be discharged to an independent housing situation. The shelter partners with agencies who provide that housing, otherwise, the shelter provides 3 months of subsidized housing with the expectation that women will secure employment within that time frame and become responsible for the rent. Women were placed in a variety of housing programs which included both abstinence- and non-abstinence based, as well as treatment contingent and non-contingent. In this study, those assigned to TAU did not receive project supported housing or the accompanying support services of CRA and case management, but received the services that they would normally receive through the community	Women were recruited from homeless shelters; the intervention took place in part at the homeless shelter and in part at the community once they had been housed	60 homeless mothers (30 EBT, 30 TAU)<br><br>Mean age 26.3, 75% African American, mean number of children 2.82	Q1: Substance use: Substance use was measured via The Form 90 Interview (Miller, 1996) at both baseline and follow-up assessments. The Form 90 is a semi-structured questionnaire that measures the frequency and quantity of drug and alcohol use in the past 90 days. Research assistants also collected urine samples from the mothers at baseline and at the 3, 6, and 9-month follow-up assessments. Mothers also self-reported problem consequences of their substance abuse by completing Inventory of Drug Use Consequence<br><br>Q2: N/A	NA	Women showed a quicker decline in alcohol use than women in treatment as usual. However, by the nine-month follow-up, the differences between conditions were not apparent. Further, all mothers showed significant improvements in problem consequences associated with alcohol and drug use and in frequency of drug use over time, with no statistically significant differences between the treatment conditions. The exploratory analysis examined the relationship between housing and substance use, showing that housing at 3 months was associated with reduced drug and alcohol use at six months and housing at 6 months was associated with reduced alcohol use at nine months. This finding supports research suggesting that housing itself is associated with reductions in substance use among those experiencing homelessness	NA

Slesnick et al. 2023 USA<br><br>RCT	To compare patterns of change in substance use and self-efficacy for young mothers randomly assigned to housing + support services (n = 80), housing-only (n = 80), and SAU (n = 80) at baseline and 3-, 6-, –9-, and 12-months post-baseline.	Housing only or TAU	Not stated	232 women<br><br>Mean age 21.57 (SD 1.80), 86.2% Black/African American, number of children M=1.59, length of current homelessness M=311.8 days (SD=470.2). 233 single, 12 cohabiting, 2 married, 2 separated and 1 divorced.	Q1: 1.Substance use measured by the Form-90<br>2. Self-efficacy measured by the 7- item Mastery Scale<br>3. Depressive symptoms measured using the Beck Depression Inventory II<br>4. Maternal history of childhood abuse measured using the demographic questionnaire at baseline<br><br>Q2: N/A	NA	Mothers showed a significant reduction in substance use in each condition (BHOU+SS = − 5.45, SE = 1.21, $p < 0.001$; BHOU = − 3.36, SE = 1.10, $p = 0.002$; BSAU = − 5.88, SE = 1.22, $p < 0.001$), and improved self-efficacy in each treatment condition (BHOU+SS = 0.66, SE = 0.12, $p < 0.001$; BHOU = 0.48, SE = 0.10, $p < 0.001$; BSAU = 0.57, SE = 0.09, $p < 0.001$). Mothers in the moderate-increasing SU (substance use) + stable SE (self-efficacy) and high-stable SU + stable SE groups showed relatively worse outcomes for substance use and self efficacy among the five trajectory groups. participants in the housing + supportive services condition showed superior outcomes by maintaining low levels of substance use and showing improved self-efficacy, while participants in the SAU condition who reported high initial levels of SU showed no improvement over time. Participants in the housing-only condition showed moderate increasing of	NA

							substance use and no self-efficacy improvement, while those in the SAU condition showed reduction of SU and improvement in SE	

Stahler et. Al 2005 USA<br><br>Non-randomised trial	This study evaluated Bridges to the Community, a supplemental component to an intensive residential treatment program. Bridges uses members of African-American churches as mentors for homeless Africa American women suffering from cocaine addiction. The primary outcome was drug use, the secondary outcomes included depression symptoms	The Hutchinson Place standard treatment was a residential treatment facility that was largely 12-step oriented. The program was intended to provide clients with transitional living for 6 months, though clients sometimes could stay as long as 12 months. Clients were provided with comprehensive services, including individual counselling, daily group counselling, case management services, relapse prevention, medical screening and care, psychiatric services, structured training groups, HIV counselling, and recreational opportunities.	Residential drug addiction treatment centre for homeless women	111 homeless women with addictions (47 in the Bridges group, 64 in the TAU group)<br><br>The clients were primarily welfare dependent African-American women who had not completed high school, and were never married. Mean age was 32.7.	Q1: Substance and alcohol abuse: Addiction Severity Index (McLellan, Luborsky, Cacciola,<br>Mental health outcomes: Beck Depression Inventory (Beck & Steer, 1987).<br><br>Q2: Client satisfaction survey (Atkisson and Zwick, 1982)		At follow-up, 100% of participants in the Bridges group reported that they had abstained from cocaine in the last 30 days compared to the standard treatment group, which reported an average of about 2 days of use. A 2 (time) x2x(group) split plot analysis of variance on mean number of days of reported drug use in the last 30 days for cocaine, alcohol, and more than one substance revealed statistically significant main effects of time and group, but no statistically significant interactions. Women in both the standard treatment and standard treatment plus Bridges reported fewer days of cocaine, F(1, 50) = 41.78, p = 0.000, alcohol, F(1,55) = 35.31, p = 0.000, and combined drug use at follow-up, F(1,58) = 10.87, p = 0.002. Drug use was completely eliminated for women who received the Bridges component, while low levels of drug use were reported by those in the standard	There were also a number of differences between groups in terms of satisfaction with services received while they were in Hutchinson Place. Virtually every satisfaction item was in the direction of being higher for the Bridges group, with a number of them being statistically significant. Specifically, compared to standard treatment clients, Bridges clients reported greater satisfaction with the comfort and attractiveness of the residential treatment program, t(56) =3.19, p = 0.002, the help that they received, t(56) =3.18, p = 0.002, staff listening skills, t(56) =4.1, p = 0.00, receiving the services that they wanted, t(56) =2.9, p = 0.005, staff competence and knowledge, t(56) =3.28, p = 0.002, and quality

							treatment only condition. The between group differences were significant; i.e., cocaine, $F(1,50) = 6.29$, $p = 0.015$, alcohol, $F(1,55) = 4.07$, $p = 0.049$, and combined substances, $F(1,58) = 7.74$, $p = 0.007$. Again, a 2 (time) x 2 (group) split plot analysis of variance on these measures revealed a statistically significant main effect of group for the Beck Depression Inventory only, $F(1,54) = 5.96$, $p = 0.018$; women in the Bridges group reported significantly less depression than women in the standard treatment only group.	of services, $t(56) = 2.1$, $p = 0.041$.

Stahler et. Al 2007 USA<br><br>RCT	This study evaluated Bridges to the Community, a supplemental component to an intensive residential treatment program. Bridges uses members of African-American churches as mentors for homeless Africa American women suffering from cocaine addiction. The primary outcome was treatment retention, secondary outcomes were treatment attendance and drug use.	All participants received a standard treatment program provided by the residential treatment facility lasting 6 -9 months. The standard treatment program provided a supervised drug-free environment with 24 hour per day staffing, room and board. It was planned as a nine-month intervention consisting of an intensive structured schedule of individual and group counselling. Because residential facilities increase participants' awareness or ditlerences in experimental groups, we were concerned that the potential for attention effects could be heightened in this setting. To control for attention effects, during the times that the Bridges group participated in contingent group activities, a special activity was provided for the control group. These activities included such things as listening to speakers address topics on employment, financial or household management, and health issues; practicing guided imaging and relaxation techniques; watching a movie of the	Residential drug addiction treatment centre for homeless women	18 women (8 Bridges, 10 control)<br><br>The mean age of the women was 32, and 78% were unmarried. All but one of the women primarily identified as being Black, non-Hispanic (94%). The remaining individual was Black, Hispanic. Mean number of years of education was 11 (range seven to 18). The primary drug of abuse was reported to be cocaine for all participants with an average of 11.5 days of use in the 30 days prior to intake. The majority of women in the sample reported being Baptist (66,7%), with 27.7% reporting no religious affiliation.	Q1: The primary outcome measure for this study was treatment retention at three and six months post-intake. Although we initially planned to conduct four quarterly follow-ups during the year after study intake, the residential treatment facility was forced to close during the study as a result of changes in funding. Treatment retention was defined as the number of participants still in treatment at three months after intake or the number of participants who had either completed six months of treatment or had transferred successfully to a new treatment program by six months after intake. Treatment attendance. We report treatment attendance as a secondary measure of treatment retention. Treatment attendance was assessed as the number of days the treatment center staff reported that the participant attended treatment up to six months after study intake.<br>Drug use. Drug use was considered a secondary measure for the purposes of this study because the residential facility limited opportunities for drug use. As such, drug use tended to occur only when a patient dropped out of the treatment program.	NA	Treatment retention. Chi- square analyses on the number of participants retained in treatment at three- and six-months post intake resulted in statistically significant differences between the Bridges and control conditions at three months and at six months post intake. More women receiving the Bridges program remained in the residential treatment program as compared to those women in the Control group at both the three-month (88% vs, 40%, respectively) and six-month (75% vs. 20%) follow-up assessments.<br>Attendance. Independent t-tests on total number of days the participants attended the residential treatment program at the end of six months approached significance. During the six-month period, all but one participant in the Bridges program attended over 100 days of treatment, compared to only half in the Control.<br>Drug use. Weekly urine samples confirmed the	NA

		participants' choice; or self-grooming activities. Most activities were conducted or arranged by the research coordinator, but educational guest speakers were used periodically. These control group activities, in contrast to the Bridges program, were not specifically designed to promote socialization or cultural identity, and did not focus on developing relationships with drug-free individuals outside of the treatment facility.			Research assistants collected urine samples for all participants once weekly on a randomly selected day and at three- and six-month post-intake assessments.<br><br>Q2: N/A		treatment facility's report that drug use was rarely detected during treatment (none of the weekly during treatment samples tested positive). As such, results are presented for the three and six-month assessments only. Follow-up rates were 88.9% and 77.8% respectively at these assessments. Chi-square analyses on urine results at three- and six-months post-intake revealed a statistical y significant difference at six months. 75% of participants in Bridges versus 30% of participants in the control group gave clean urines. Although not statistically significant more participants in the Bridges condition gave clean urines at three months (M= 88%) than participants in the control condition. Most of the drug positive urines detected were positive for cocaine (85 .7%); the rest were positive for marijuana only (14.3 %).	

Upshur et al. 2015 USA<br><br>RCT	To evaluate the Collaborative Care Model (CCM) in a small pilot, clinician randomized trial, to treat women who screened positive for hazardous drinking during a primary health care visit in a health care for the homeless clinic. Primary outcome was initiation, engagement and retention in alcohol treatment services.	Usual care patients did not receive<br>referrals to, or outreach from, the study-trained CM and their PCPs were not provided any<br>alcohol intervention training or patient materials. They delivered usual care for medical conditions, including any behavioral health or drug or alcohol use problems.	Primary care clinic for homeless people	15 clinicians were randomised to be trained in the CCM intervention or to continue to deliver usual care (7 CCM, 8 usual care). Patients had an existing clinician, and therefore the group assignment of a woman depended on the random assignment of her clinician (42 CCM, 40 usual care)<br><br>Participants were on average in their mid-40s,<br>had multiple recent housing situations including in their own apartment, on the street, in shelters, or (data not shown in table) staying with relatives or friends, in hotels/motels, detox, in jail, hospitalized for medical or mental health conditions, in medical respite care,<br>or group homes. Women were 41% Black, and 33% white, with the remainder of multiple races, Latino, or unknown. Only one participate was Asian.	Q1: Reduction in alcohol and drug use: The Time Line Follow Back (Sobell and Sobell 1992, Sacks, Drake, Williams, Banks, Herrell, 2003) was used to determine monthly alcohol consumption in the last three months. The same procedure was used to determine the number of days in the last three months that illegal drugs were consumed<br>Alcohol use consequences: A self-report measure developed by Weisner and Schmidt, 1995<br>collected information on 11 items regarding the negative consequences of alcohol use<br>Mental and physical health status: General mental and physical health status was measured using the SF-8 (Ware, Kosinski, Dewey, & Gandek, 2001).<br><br>Q2: N/A	NA	The rate of alcohol consumption decreased dramatically at both 3- and 6-month follow-up time points for both groups, from a median of 185 drinks/month at baseline, to 12 drinks/month for the intervention group and from a median of 87.3 drinks/month to 1.3 drinks/month for the usual care group, at 6-months. For both groups this represented a significant reduction in alcohol consumption (p=<.001), but we found no statistical differences between groups. There were no significant differences between groups at follow-up in overall mental or physical health	NA

Weinreb et al. 2016 USA<br><br>Cohort study of two different family shelters	Evaluate a new intervention, the Integrated Care Model for Homeless Mothers (ICMHM), adapted from the collaborative care model to address unique aspects of care for homeless mothers who screened positive for depression during a shelter-based primary care visit. Primary outcome was depression severity	At the usual-care site, women who screened positive for depression also were provided appointments with the PCP who initiated treatment as usual, which could include antidepressant medication and recommendation for psychotherapy outside the clinic. Women in the usual-care group received general case management services that were available to all families receiving health services at the clinic. These services included, for example, assistance with obtaining public benefits, linking with community resources for family activities, outside mental health or substance use services, and meeting children's educational needs.	Family homeless shelters	67 homeless women (42 at intervention shelter, 25 at control shelter)<br><br>Women in both conditions were similar in all characteristics except for number of children (P = .01) and SF-8 physical health score (P = .02). Women were on average 36 years of age (intervention: 35.2 years, usual care: 38 years); 52.2% had been homeless before. The majority of women were on Medicaid (intervention: 85.4%, usual care: 80%). Almost 100% of women in both groups reported a past history of childhood or adulthood abuse (intervention: 97.3%, usual care: 100%). Women reported an average of 3 chronic illnesses (intervention: 2.5, usual care: 2.7).	Q1: The Hopkins Depression Symptom Checklist-Depression Scale 2019,20 was used to confirm depression symptoms at baseline and to document the primary outcome of reduction in depressive symptoms at follow-up.<br>Additional measures used to evaluate comorbidities and possible confounders included the Posttraumatic Diagnostic Scale22 to determine stress related to violence and other threatening events at baseline, the 10-item Drug Abuse Screening Test23,24 to ascertain misuse of prescription or street drugs and the Alcohol Use Disorders Identification Test-Consumption25 to screen for comorbid alcohol use problems at baseline and follow-up, the Medical Outcomes Study 8-item Short-Form Health Survey (SF-8)26 to ascertain general physical and mental health at baseline and follow-up, and the 7-item Generalized Anxiety Disorder scale27 to identify generalized anxiety at baseline and follow-up.<br><br>Q2: N/A	NA	We observed a significant drop in the continuous depression score for all women by the end of follow-up (−0.27, 95% CI = −0.50 to −0.05, P = .02 for time trend). There were no significant differences detected between study groups in the continuous depression outcome measure at follow-up; however, the difference in the proportion of women achieving ≥ 50% improvement in depression symptoms at 6 months approached significance (intervention: 30%, usual care: 5.9%, P = .07). Self-report of receiving current depression treatment differed significantly between groups at 3 months (intervention: 60%, usual care: 20%, P = .01) and 6 months (intervention: 40%, usual care: 5.9%, P = .01). Women in the intervention group also had significantly more PCP and care manager visits at both 3-month (74.3% vs 53.3% with ≥ 2 PCP visits, P = .009; 91.4% vs 26.7% with ≥ 2 care manager visits, P < .001, respectively) and	NA

							6-month (46.7% vs 23.5% with ≥ 2 PCP visits, P = .003, respectively); 70% vs 17.7% with ≥ 2 care manager visits, P = .001, respectively) follow-up than women in the control group. Further, by 6-month follow-up, 73.3% of women in the intervention group had been prescribed an antidepressant medication compared to 5.9% of women in the usual-care group (P ≤ .001).<br>There were no significant differences between groups in anxiety, mental or physical health functioning,	
