# Supplementary material for: Interventions to improve the mental health of women experiencing homelessness: A systematic review of the literature
Source: PLoS One. 2024 Apr 3;19(4):e0297865. doi: 10.1371/journal.pone.0297865 (PMC10990227; doi:10.1371/journal.pone.0297865)
Supplement: S3 File — (PDF) [file pone.0297865.s004.pdf]

Study	Population	Number of participants	Country and recruitment setting	Study design	Intervention type	Comparator type	Outcome measures
**Bain, 2014**	Mothers with infants residing in homeless shelters	22	South Africa, 2 homeless shelters in Johannesburg	RCT	Therapy	Control group: shelter services as usual	Depression and anxiety measured on the Kessler-10 scale
**Bani-Fatemi et al., 2020**	Women aged 16-24 experiencing homelessness and gender-based violence	70	Canada, community resource centre and shelter for homeless youth in Toronto	Uncontrolled before-and-after study	Therapy	Pre-intervention period	1. Psychological health measured by the WHO-QOL (psychological health domain) score.<br>2. Anxiety measured by HADS-A scale<br>3. Depression measured by HADS-D scale<br>4. PTSD measured by UCLA-PTSD Scale
**Castanos-Cervantes et al., 2019**	Girls aged 9-17 experiencing homelessness	84	Mexico, homeless shelters in Mexico City	Quasi-experiment	Therapy	Control group: shelter services as usual	Depression and anxiety measured on validated scales developed by the authors
**Constantino et al., 2005**	Women residing in domestic violence shelters	24	USA, domestic violence shelter in Western Pennsylvania	RCT	Social support intervention	Control group: shelter services as usual plus unstructured discussion session	Psychological distress measured using BSI.
**Desai et al., 2008**	Homeless female veterans who were part of the Homeless Women Veterans Programme	643	USA, Veterans' Affairs medical centres across the country	Controlled before-and-after study	Therapy	Control group: services as usual from Homeless Women Veterans' Programme	1. Psychological health measured by SCL-30-R score and SF-12 Score (Mental subscale)<br>2. Alcohol and drug abuse measured by ASI score<br>3. PTSD measured by PCL score
**Grabbe et al., 2013**	Homeless women who used a daytime homeless shelter	8	USA, daytime homeless shelter in a large South-Eastern city	Qualitative study	Personal / skill development programme	-	Acceptability of intervention was determined through semi-structured interviews
**Graziano et al., 2023**	Children 18 months-5-year-old and their mothers	144 mother-child dyads	USA, women's homeless shelter	Pilot RCT	Therapy		1.Acceptability and feasibility measured by intervention completion and attendance, and consumer satisfaction<br>2. Parenting stress measured using Dyadic Parent–Child Interaction Coding System–4th Edition<br>3. Child externalising behaviours measured with Eyberg Child Behavior Inventory (ECBI)

							4. Posttraumatic stress symptoms measured with the Child and Adolescent Trauma Screen– Caregiver (CATS-C)
**Guo et al., 2012**	Mothers with children residing in homeless shelters	60	USA, homeless families shelter	RCT	Multi-factorial intervention	Control group: shelter services as usual	1. Psychological health measured by SF-36 Score (Mental subscale)<br>2. Depression measured by BDI-II
**Harpaz-Rotem, 2011**	Homeless female veterans	451	USA, Veterans' Affairs medical centres across the country	Controlled before-and-after study	Multi-factorial intervention	Control group: services as usual from Veterans' Affairs medical centres, did not utilise >30 days of residential treatment	1. Psychological health measured by SCL-30 Score and SF-12 Score (Mental subscale)<br>2. Alcohol and drug abuse measured by ASI score<br>3. PTSD measured by PCL score
**Hernandez-Ruiz et al., 2005**	Women residing in domestic violence shelters	28	USA, 2 domestic violence shelters in an upper Mid-Western city	Quasi-experiment	Relaxation	Control group: relaxation in quiet room without music	Anxiety measured by STAI score
**Herschell et al., 2017**	Mothers residing in a domestic violence shelter	17	USA, domestic violence shelter	Uncontrolled before-and-after study	Parenting intervention	Pre-intervention period	1. Psychological health measured by SCL-90-R score<br>2. Acceptability measured by Barriers to Treatment Participation Scale and Therapy Attitude Inventory
**Johnson & Zlotnick, 2006**	Women residing in domestic violence shelters	18	USA, 2 domestic violence shelters	Uncontrolled before-and-after study	Therapy	Pre-intervention period	1. Depression measured by BDI Score<br>2. PTSD measured by CAPS Score<br>3. Acceptability measured by Client Satisfaction Questionnaire
**Johnson et al., 2011**	Women residing in domestic violence shelters	70	USA, 2 domestic violence shelters in a mid-sized Mid-Western city	RCT	Therapy	Control group: shelter services as usual	1. Depression measured by BDI Score<br>2. PTSD measured by CAPS Score<br>3. Acceptability measured by Client Satisfaction Questionnaire and Treatment Credibility Questionnaire
**Johnson et al., 2016**	Women residing in domestic violence shelters	60	USA, 4 regional domestic violence shelters in the Midwest	RCT	Therapy	Control group: shelter services as usual	1. Depression measured by BDI Score<br>2. PTSD measured by CAPS Score<br>3. Acceptability measured by Client Satisfaction Questionnaire

**Johnson et al., 2020**	Women residing in domestic violence shelters	172	USA, 6 regional domestic violence shelters in the Midwest	RCT	Therapy	Control group: shelter services as usual	1. Depression measured by CES-D Score<br>2. PTSD measured by CAPS Score<br>3. Acceptability measured by Client Satisfaction Questionnaire
**Jourilees et al., 2009**	Mothers with 4-9-year-old children residing in domestic violence shelters	66	USA, 6 domestic violence shelters	RCT	Multifactorial intervention	Control group: social support without parenting intervention	Psychological distress measured by SCL-90-R Score
**Kahan et al., 2019**	Women aged 16-24 experiencing homelessness and gender-based violence	23	Canada, community resource centre for homeless youth in Toronto	Qualitative study	Personal / skill development programme	-	Acceptability was determined through semi-structured interviews
**Kim & Kim, 2001**	Women residing in domestic violence shelters	60	South Korea, 2 domestic violence shelters	Quasi-experiment	Therapy	Control group: shelter services as usual	1. Depression measured by CES-D Score<br>2. Anxiety measured by STAI Score
**Lako et al., 2018**	Women residing in domestic violence shelters	136	The Netherlands, 19 domestic violence shelters	RCT	Case-management	Control group: shelter after-care as usual, including follow-up meetings	1. Psychological health measured using the BSI Score<br>2. Depression measured using the CES-D Score<br>3. PTSD measured using the Impact of Event Scale
**Mallory et al., 2022**	Young mothers experiencing homelessness	240	USA, drop-in centre for homeless youth and through adds at shelters and agencies serving youth experiencing homelessness.	RCT	Multifactorial intervention	Control group: (1) housing only and (2) SAU – referral sheet including all services available in the community	1.Substance use measured using the Form-90<br>2.Depressive symptoms measured by Beck Depression Inventory II
**Marin et al., 2021**	Women residing in homeless shelters	54	Spain, 4 homeless shelters in Madrid	Uncontrolled before-and-after study	Therapy	Scores were compared at multiple time-points throughout the intervention	Acceptability measured using the Participant Satisfaction survey for satisfaction and perceived usefulness.
**Noh et al., 2018**	Women aged 12-24 years old residing in homeless shelters	32	South Korea, 5 homeless shelters	Controlled before-and-after study	Personal / skill development programme	Control group: shelter services as usual	1. Depression measured using the BDI-II<br>2. PTSD measured using the BAI

							3. Problem drinking measured using the Audit-C Questionnaire
**Nyamathi et al., 1998**	Homeless women	241	USA, community centres in Los Angeles	RCT	Personal / skill development programme	Control group: AIDS education programme without coping enhancement component	1. Psychological health measured by MHI-5<br>2. Depression measured by CES-D Scale
**Nyamathi et al., 2017**	Homeless female parolees / probationers	130	USA, shelters and homeless services in Los Angeles	RCT	Therapy	Control group: received health promotion programme instead of DBT	Alcohol and drug use measured by TCU-II form and urine toxicology
**O'Campo et al., 2023**	Women experiencing homelessness and mental illness	653	Canada, homeless shelters	RCT	Multifactorial intervention	Control group: TAU – access to housing and services through other community programmes.	1.Quality of life measured by The Lehman Quality of Life Interview 20<br>2. Community functioning measure by Multnomah Community Ability Scale (MCAS)<br>3.Psychiatric symptoms measured using The Colorado Symptom Index (CSI)<br>4. Self-reported integration with the immediate community measured by The Community Integration Scale (CIS)<br>5. Past month substance-related problems measured by Global Assessment of Individual Needs Short Screener (GAIN-SS)<br>6. Stability of housing measured by calculating the percentage of days stably housed during past 24 months.
**Rodriguez-Moreno et al., 2022 and 2023**	Homeless women residing in homeless shelters	81	Spain, public homeless shelters in Madrid	Single-blinded quasi-experimental clinical trial	Therapy	Control group: shelter services as usual	1.Anxiety and depression symptoms measured by Beck Anxiety Inventory (BAI) and Beck Depression Inventory-II (BDI-II)<br>2. Emotional functioning measured by Positive and Negative Affect Scale (PANAS)<br>3. Integrative wellbeing measured by the Pemberton Happiness Index (PHI)

							4. Health status was measured by the Short Form Health Survey (SF-12<br>5. Social support was measured by the Social Support Questionnaire (SSQ6)<br>6. Anxiety and depression severity 7. Functional impairment, measured with the Overall Anxiety Severity and Impairment Scale (OASIS) and the Overall Depression Severity and Impairment Scale (ODSIS)
**Rodriguez-Moreno et al., 2020**	Women residing in homeless shelters	81	Spain, homeless shelters in Madrid	Quasi-experiment	Therapy	Control group: shelter services as usual	1. Depression measured by BDI-II and ODSIS scale<br>2. Anxiety measured by BAI and OASIS scale
**Sacks et al., 2004**	Homeless mothers with clinical substance abuse	196	USA, 4 residential drug treatment centres in Pennsylvania	Quasi-experiment	Multifactorial intervention	Control group: traditional residential therapeutic community methods	1. Psychological distress measured as a composite of ASI, BDI and SCL-90-R<br>2. Substance abuse measured as a composite of measures of frequency, type and impact of use
**Salem et al., 2017**	Pre-frail and frail homeless women	32	USA, homeless day centre in Los Angeles	RCT	Personal / skill development programme	Control group: health promotion intervention	Substance abuse measured using the TCU form
**Samuels et al., 2015**	Mothers with mental / substance abuse problems residing in domestic violence shelters with 18 month – 16 year old children in their care	210	USA, family homeless shelter in New York State	RCT	Case management	Control group: shelter services as usual	Psychological health measured using the BSI
**Shors et al., 2014**	Mothers residing in a homeless shelter	14	USA, homeless shelter	Quasi-experiment	Multifactorial intervention	Control group: shelter services as usual	1. Depression measured by BDI<br>2. Anxiety measured by BAI
**Slesnick & Erdem, 2012**	Mothers with clinical substance abuse with 2-6 year old children residing in homeless shelter	15	USA, family homeless shelter in a large Midwestern city	Uncontrolled before-and-after study	Multifactorial intervention	Pre-intervention period	1. Psychological health measured by SF-36v2<br>2. Depression measured by BDI-II<br>3. Substance abuse measured by Form 6 drug and alcohol interview and urine toxicology

**Slesnick & Erdem, 2013**	Mothers with clinical substance abuse with 2-6 year old children residing in homeless shelter	60	USA, family homeless shelter	RCT	Multifactorial intervention	Control group: shelter services as usual	Substance abuse was measured by the Form 90 Interview, Inventory of Drug Use Consequence and urine toxicology
**Slesnick et al., 2023**	Women between 18 - 24-year-old, experiencing homelessness and with a substance use disorder (SUD) who have biological child under <6 in their care.	240	USA, homeless service agencies and adds in shelters, drop-in centres and other agencies serving homeless youth, in a large Midwestern city	RCT	Multifactorial intervention	Control groups: (1) housing only, (2) SAU – services normally offered in the community	1.Substance use measured by the Form-90<br>2. Self-efficacy measured by the 7- item Mastery Scale<br>3. Depressive symptoms measured using the Beck Depression Inventory II<br>4. Maternal history of childhood abuse measured using the demographic questionnaire at baseline
**Stahler et al., 2005**	Homeless mothers who abused cocaine residing in substance abuse residential treatment centre, with pre-school children in their care	111	USA, residential substance abuse treatment centre in Philadelphia	Quasi-experiment	Multifactorial intervention	Control group: residential substance abuse rehabilitation services as usual	1. Depression was measured by BDI<br>2. Substance abuse was measured by ASI<br>3. Acceptability was measured by Client Satisfaction Survey
**Stahler et al., 2007**	Homeless women <18 years of age who abused cocaine residing in substance abuse residential treatment	18	USA, residential substance abuse treatment centre	RCT	Multifactorial intervention	Control group: residential substance abuse rehabilitation services as usual	Substance abuse was measured by urine toxicology
**Upshur et al., 2015**	Homeless women who screen positive for hazardous drinking	82	USA, health centre in the North-East	RCT	Collaborative Care Model	Control group: received health care from primary care physicians not trained in the collaborative care model	Substance abuse measured by Time Line Follow Back and a validated self-report measure of alcohol use consequences
**Weinreb et al., 2016**	Mothers residing in homeless shelters who screen positive for depression	67	USA, 2 homeless shelters in New York City	RCT	Collaborative Care Model	Control group: shelter and medical services as usual	Depression was measured using the Hopkins Depression Symptom Checklist Depression Scale
