# Supplementary material for: Interventions to improve the mental health of women experiencing homelessness: A systematic review of the literature
Source: PLoS One. 2024 Apr 3;19(4):e0297865. doi: 10.1371/journal.pone.0297865 (PMC10990227; doi:10.1371/journal.pone.0297865)
Supplement: S4 File — (PDF) [file pone.0297865.s005.pdf]

Effects of interventions on selected mental health outcomes

Figure 1: Effectiveness of different intervention categories in improving depression outcomes

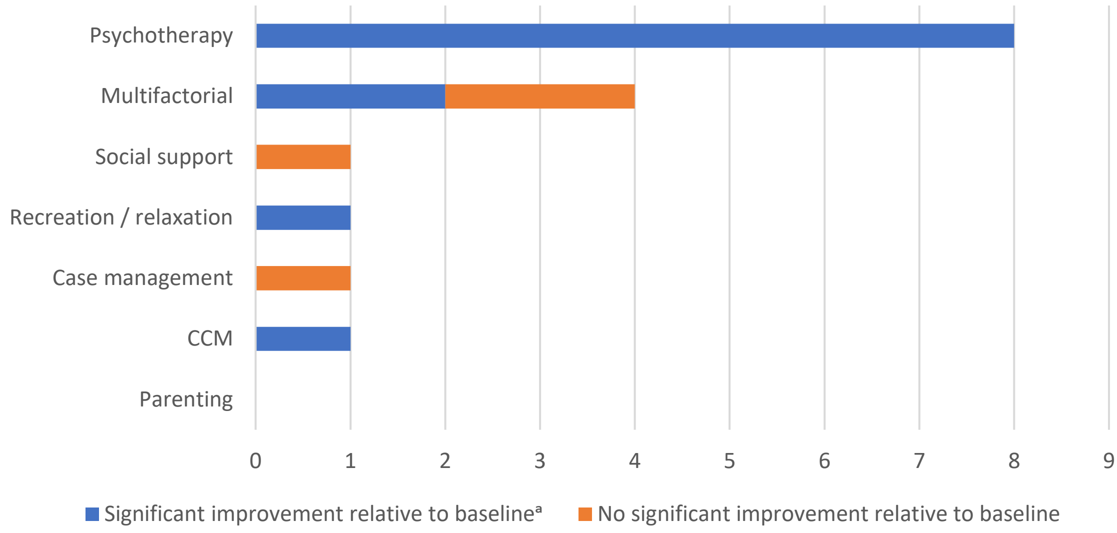

[a] Intervention group shows significant improvement in ≥1 measure of depression from baseline to ≥1 subsequent time points

Figure 2: Effectiveness of different intervention categories in improving anxiety outcomes

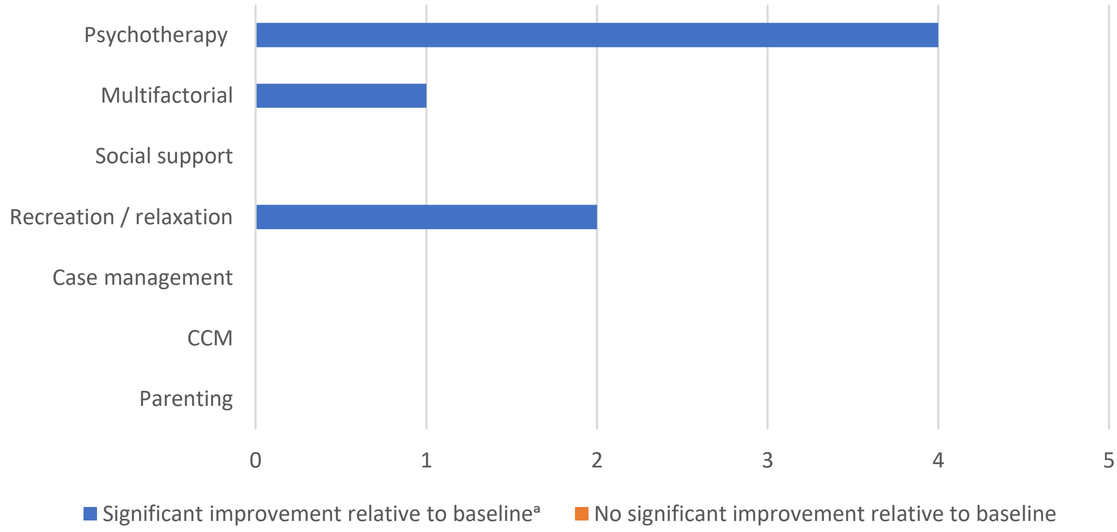

[a]Intervention group shows significant improvement in ≥1 measure of anxiety from baseline to ≥1 subsequent time points

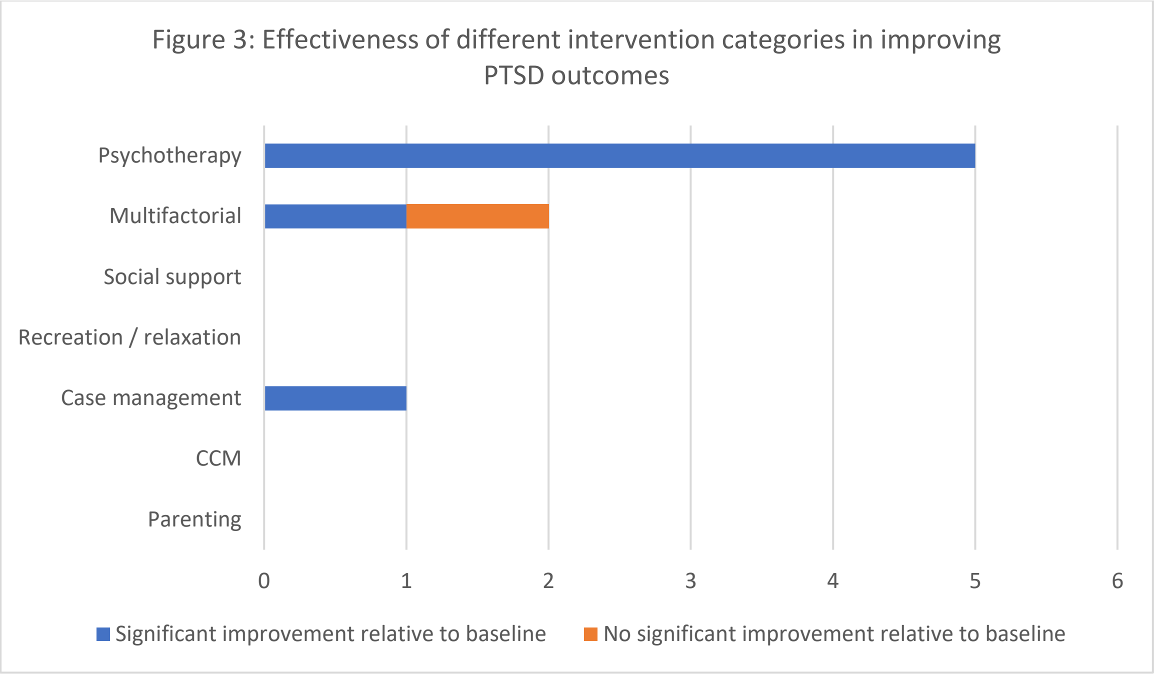

[a]Intervention group shows significant improvement in ≥1 measure of PTSD from baseline to ≥1 subsequent time points

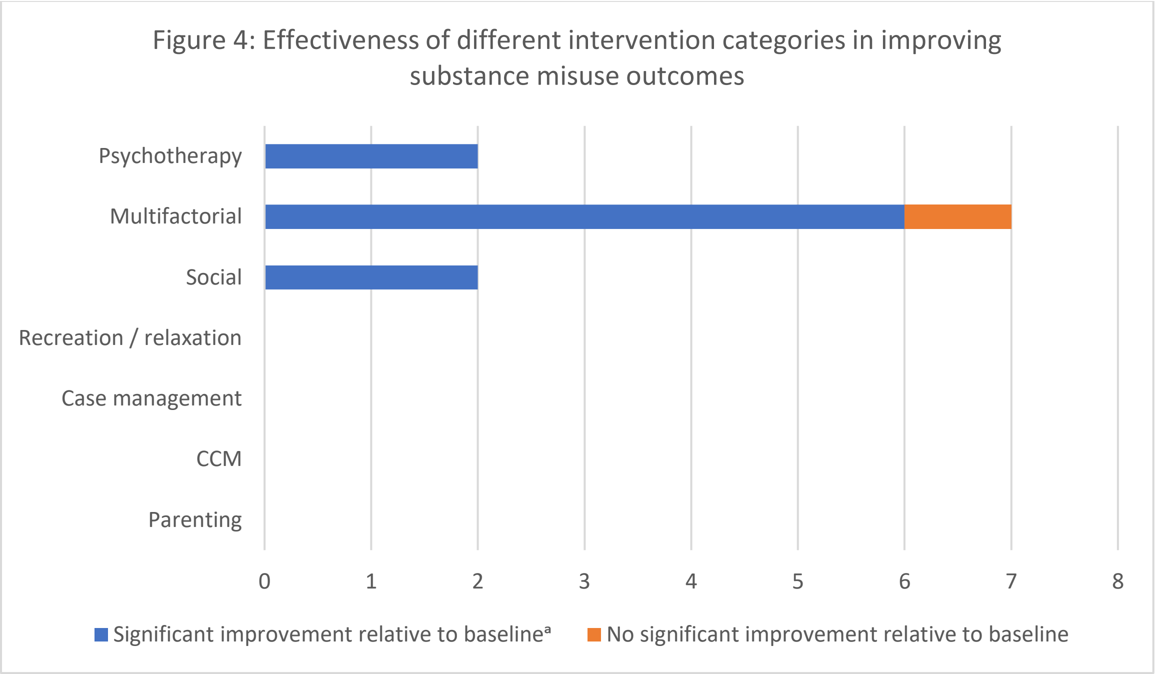

[a]Intervention group shows significant improvement in ≥1 measure of substance misuse from baseline to ≥1 subsequent time points

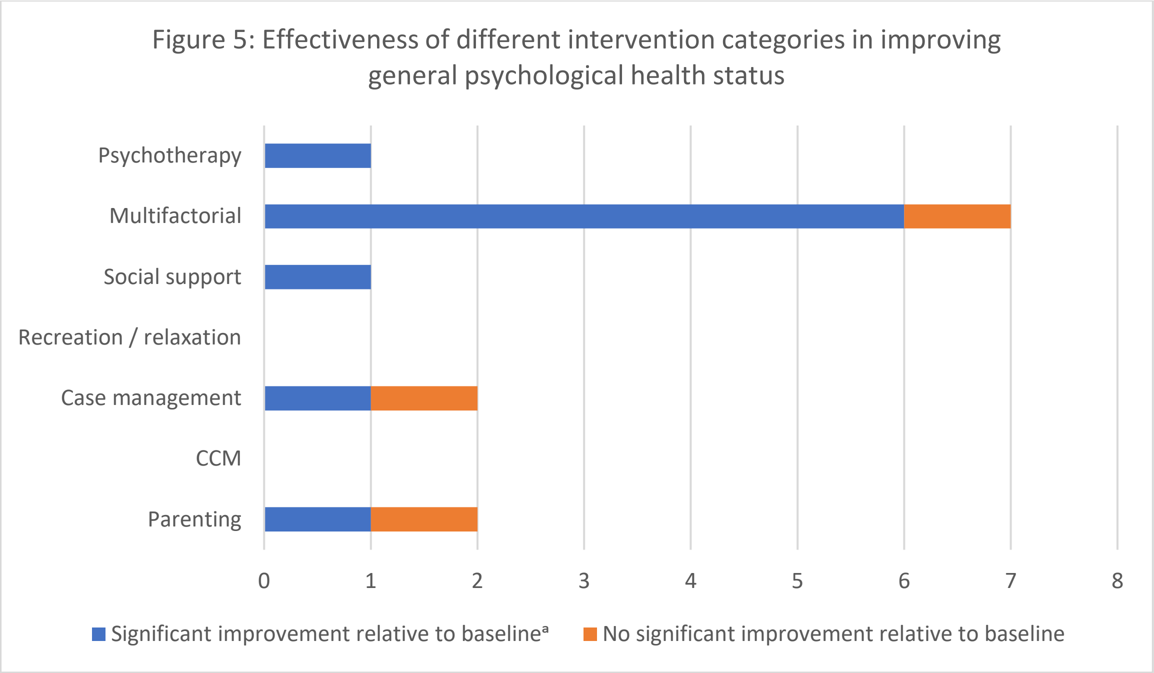

[a]Intervention group shows significant improvement in ≥1 measure of psychological health status from baseline to ≥1 subsequent time points
