# Supplementary material for: Interventions to improve the mental health of women experiencing homelessness: A systematic review of the literature
Source: PLoS One. 2024 Apr 3;19(4):e0297865. doi: 10.1371/journal.pone.0297865 (PMC10990227; doi:10.1371/journal.pone.0297865)
Supplement: Attachment — Submitted filename: Review of PONE-D-23-33329.docx [file pone.0297865.s006.docx]

Review of PONE-D-23-33329

This systematic review examines the state of the literature focused on reducing mental health disparities among women experiencing homelessness. The review appears to have been conducted with rigor and authors offer a thorough account of their processes. Findings are well organized and effective for readers. Overall, the review is effective and offers a helpful contribution to the literature in its summation. I have a few minor comments listed below.

- Unless I missed it, I saw no date range included in the inclusion criteria. Articles were published between 1998 and 2023, but what range was specified in the searches?
- Inclusion criteria specified studies were to consist of >90% girls/women. How many were focused solely on the population of interest and how many were a mixture of genders, where women were >90% of the sample?
- Table 4 is very effective in communicating the qualities of the studies. However, I am interested in the definitions of the classifications in the EPHPP Quality Assessment Tool. For example, what indicates a study has moderate selection bias? Is there a way to effectively communicate these definitions?
- The structure of the results is quite effective in communicating findings. Most readers will likely be interested in identifying a best practice for a specific condition in this population and authors have effectively done that.
