# Supplementary material for: Interventions to improve the mental health of women experiencing homelessness: A systematic review of the literature
Source: PLoS One. 2024 Apr 3;19(4):e0297865. doi: 10.1371/journal.pone.0297865 (PMC10990227; doi:10.1371/journal.pone.0297865)
Supplement: Attachment — Submitted filename: Response to reviewers_PONE-D-23-33329_050124.docx [file pone.0297865.s007.docx]

**Review of PONE-D-23-33329**

05.01.2024

Dear Academic Editor and Reviewers,

Thank you for your helpful comments regarding out research article:- “Interventions to improve the mental health of women experiencing homelessness : A systematic review of the literature”.

We have addressed each of the reviewer’s points in the table below and highlighted the changes in the main manuscript using tracked changes. We believe the manuscript has greatly improved based on the reviewers’ feedback.

Thank you for your consideration of this manuscript.

Sincerely,

Anne-Marie Burn (Corresponding author)

| Reviewer #1 | Authors’ response |
| --- | --- |
| This systematic review examines the state of the literature focused on reducing mental health disparities among women experiencing homelessness. The review appears to have been conducted with rigor and authors offer a thorough account of their processes. Findings are well organized and effective for readers. Overall, the review is effective and offers a helpful contribution to the literature in its summation. I have a few minor comments listed below. | Thank you for your helpful feedback. |
| Unless I missed it, I saw no date range included in the inclusion criteria. Articles were published between 1998 and 2023, but what range was specified in the searches? | We have included studies from the earliest available. It is listed in Table 1: Inclusion criteria which we have highlighted in yellow. |
| Inclusion criteria specified studies were to consist of >90% girls/women. How many were focused solely on the population of interest and how many were a mixture of genders, where women were >90% of the sample? | All included studies focused solely on the population of interest; no studies included homeless men. We have now clarified this in the text (Participant characteristics section, p10). |
| Table 4 is very effective in communicating the qualities of the studies. However, I am interested in the definitions of the classifications in the EPHPP Quality Assessment Tool. For example, what indicates a study has moderate selection bias? Is there a way to effectively communicate these definitions? | We have added rows to the Table 4 to explain how ratings are decided for each criterion of the tool. |
| The structure of the results is quite effective in communicating findings. Most readers will likely be interested in identifying a best practice for a specific condition in this population and authors have effectively done that. | Thank you! |
| Reviewer #2 |  |
| This systematic review aimed to examine the effectiveness and acceptability of interventions aimed to improve the mental health of homeless girls and women. Abstract: clear and concise. | Thank you for your feedback – we appreciate it. |
| Introduction: A brief description of ‘rough sleepers’ on page 3 may help as I do not believe this term is used outside the UK. I had to look it up. Overall, introduction is clear and concise. It makes a strong argument for the systematic review in order to address this important issue. | Thank you for bringing it to our attention. We have added an explanation where we first introduced the term (p3). |
| Aims and Research Questions: The word ‘question’ is missing the ‘s’ | We have corrected it. |
| Methods: appear appropriate. Search Strategy and selection criteria: a short sentence on what the PICO framework is and regularly used for would be helpful. | We have added a sentence to clarify the rational for using PICO criteria (p5). |
| Inclusion Criteria: clarify whether any age was included (it appears so but just be explicit) | We have clarified in Table one (Population) that indeed studies with female participants of any age are eligible for inclusion. |
| Study selection: in those articles that were double reviewed, how much agreement/disagreement was there? I don't believe an exact percent agreement necessary but a broad statement would be helpful. | We have included kappa values for interrater agreement at both title/abstract and full text screening stages (p7). |
| Pg. 11 Psychotherapy interventions; reword/phrase the last two sentences, they read poorly. Possible alternative: “In all studies, psychotherapy was delivered in person, whether in a group context (n=6), individual (n=4) or a mixture of group and individual sessions (n=1). The most commonly use therapeutic model was cognitive behavioral therapy (CBT). In addition, dialectical behavioral therapy, resilience enhancement therapy, and crisis intervention were used." | Thank you, we have now reworded both sentences. |
| Study quality: more detail on the CASP checklist needed; what was the criteria – possibly present in a table | We have added a couple of sentences explaining the CASE tool in the Methods section (critical appraisal, p7) |
| Results - if you are able to come up with a table to represent the effectiveness of the studies, that may be helpful to summarize your findings. If you could develop a table that doesn't take up more than a page I would add it, but if not, it probably wouldn't add anything to your article. The way you present them are clear but hard to get a good overall sense of what you found. | We have included a new table (Table 5) and have produced graphs (see supplementary file) showing the number of interventions that produced statistically significant improvement in selected mental health outcomes. |
| Top of pg. 35 – references in last paragraph need fixing Top of pg. 37 - 39 – extra line between paragraphs in a number of places | We have now addressed it. |
| Discussion: Pg. 44 – first sentence of second paragraph. Saying that substance misuse ‘deteriorated’ is not clear to me, I had to re-read this sentence a few times. Reword to make clearer. I believe you are saying that effectiveness of substance misuse programs waned over time with an increase in substance use in follow-up | We have reworded the sentence to make it clearer. |
| Conclusions: You mention that homeless women without children and elderly homeless women have unique vulnerabilities and needs that should be addressed, which is certainly true, but I think a particularly unique issue some women face is those who may be pregnant or mothers – they have the added stress of worrying about the child(ren), may face more difficulties in securing adequate housing, and may be more likely to stay in unsafe environments in order to keep a roof over their child(ren). This is very briefly mentioned in the introduction but a few sentences either added to the introduction or conclusions would strengthen your article. | We agree that this is an important point and we have added a few sentences in Conclusions to address this. |
| Why do you think only studies from US and Canada (except one) recorded ethnicity? | This sentence contains an error and has been corrected. 6 studies conducted in the USA did not record ethnicity. |
